# Rapid Production of Metal–Organic Frameworks Based Separators in Industrial‐Level Efficiency

**DOI:** 10.1002/advs.202002190

**Published:** 2020-11-06

**Authors:** Guang‐Kuo Gao, Yi‐Rong Wang, Hong‐Jing Zhu, Yifa Chen, Ru‐Xin Yang, Cheng Jiang, Huiyuan Ma, Ya‐Qian Lan

**Affiliations:** ^1^ School of Materials Science and Engineering College of Chemical and Environmental Engineering Harbin University of Science and Technology Harbin 150040 China; ^2^ Jiangsu Collaborative Innovation Centre of Biomedical Functional Materials Jiangsu Key Laboratory of New Power Batteries School of Chemistry and Materials Science Nanjing Normal University Nanjing 210023 China; ^3^ Changzhou Institute of Innovation and Development Nanjing Normal University Nanjing 210023 China; ^4^ School of Chemistry South China Normal University Guangzhou 510006 P. R. China

**Keywords:** industrial‐level efficiency, lithium–sulfur battery separators, MOF‐based mixed matrix membranes

## Abstract

Metal–organic framework (MOF) based mixed matrix membranes (MMMs) have received significant attention in applications such as gas separation, sensing, and energy storage. However, the mass production of MOF‐based MMMs with retained porosity remains a longstanding challenge. Herein, an in situ heat‐assisted solvent‐evaporation method is described to facilely produce MOF‐based MMMs. This method can be extended into various MOFs and polymers with minimum reaction time of 5 min. Thus‐obtained MMMs with high uniformity, excellent robustness, well‐tuned loading, and thickness can be massively produced in industrial‐level efficiency (≈4 m in a batch experiment). Furthermore, they can be readily applied as powerful separators for Li–S cell with high specific capacity (1163.7 mAh g^−1^) and a capacity retention of 500.7 mAh g^−1^ after 700 cycles at 0.5 C (0.08% fading per cycle). This work may overcome the longstanding challenge of processing MOFs into MMMs and largely facilitate the industrialization process of MOFs.

## Graphical abstract

An in situ heat‐assisted solvent‐evaporation method is developed to facilely fabricate porous and robust metal–organic framework‐based mixed matrix membranes (MMMs). The obtained MMMs with high uniformity, excellent robustness, well‐tuned loading, and thickness can be massively produced (≈4 m in a batch experiment in lab scale) and exhibit high performances in separators for Li–S battery.

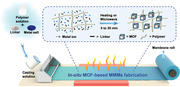

## 1 Introduction

In modern society, separation processes have accounted for about 10–15% of the total world energy consumption.^[^ ^1^ ^]^ Membrane materials are vital for separation processes and have been regarded as the bottlenecks for the development of many important fields to meet the requirements of energy saving, low‐grade raw material recycling and environmental protection.^[^ ^2^ ^]^ As a kind of important membrane materials, polymer membranes dominate the present separation market due to their relatively low cost, good processbility, high mechanical strength, and decent separation properties yet they still possess inherent compromise between permeability and selectivity.^[^ ^3^, ^4^ ^]^ To conquer it, various functional materials such as zeolites, porous carbon and metal–organic frameworks (MOFs) have been processed into polymer membranes to serve as an alternative strategy.^[^ ^5^ ^]^ Among them, MOFs preparing from rigid multitopic organic linkers and inorganic secondary building units (SBUs) have received widespread attention yet their inherent crystalline and fragile nature have largely limited the industrial processes.^[^ ^6^, ^7^, ^8^ ^]^ To solve it, MOF based mixed matrix membranes (MMMs) that can combine the good processability of polymer membranes to conquer the inherent nature of MOFs have been intensively investigated in various applications such as gas/liquid separation,^[^ ^9^ ^]^ catalysts,^[^ ^10^, ^11^ ^]^ photovoltaic cells,^[^ ^12^, ^13^ ^]^ sensing,^[^ ^14^ ^]^ and energy storage.^[^ ^15^ ^]^ The fabrication of them can be mainly achieved through in situ growth of MOFs on polymer matrices or direct mixing presynthesized MOFs with polymers followed by treatment such as casting or electrospinning.^[^ ^16^, ^17^ ^]^ In situ growth method (e.g., solvothermal synthesis) possesses high dispersity of MOFs and good compatibility between MOFs and polymers yet it generally requires a large amount of solvent, time or energy and MOF particles are only deposited on the surface of polymer matrices. For the method of direct mixing MOFs with polymers, although it can tightly integrate MOFs in the polymer system, the presynthesis of MOFs is a tedious work and there are still some inevitable problems such as the dispersity of MOF particles, the compatibility between MOF and polymers, and the percentage of remained porosity.^[^ ^18^, ^19^ ^]^ Besides, the mass production of MOF‐based MMMs with retained porosity has been regarded as a longstanding challenge for both of them. Methods that can combine the advantages of these two methods (i.e., in situ growth and direct mixing method) to massively produce MOF‐based MMMs are highly desirable yet largely unmet and have limited the development of many important fields.

As an important application field of MOF‐based MMMs, the separators for Li–S battery have drawn much attention due to the prosperously development of energy storage techniques.^[^ ^20^, ^21^, ^22^ ^]^ Specially, a Li–S separator with excellent performance needs to satisfy the properties such as: 1) certain pore size to achieve high ionic conductivity and barrier properties to block the shuttling effect of polysulfides;^[^ ^23^, ^24^ ^]^ 2) high chemical and electrochemical stability that can be resistant to the electrolyte corrosion;^[^ ^25^ ^]^ 3) good wettability of the electrolyte; 4) high mechanical or thermal stability that enable to provide automatic shutdown protection, and 5) most importantly, the possibility in mass production to meet practical applications.^[^ ^26^ ^]^ MOF‐based MMMs, combing the advantages of MOFs with polymers have much potential in the applications of Li–S separators.^[^ ^27^ ^]^ In detail, the following properties of MOFs might be vital in this field: 1) owing to the plentiful cavity structure, MOFs can effectively accommodate a variety of liquid molecules like electrolyte solution, thus MOFs with properly tuned pore sizes can be introduced as ionic sieves in battery to realize low internal resistance and high ionic conductivity;^[^ ^28^ ^]^ 2) due to the tunability of pore size, it is possible to block the passage of polysulfides through the size barrier, thereby increasing the diffusion resistance of polysulfides,^[^ ^29^ ^]^ and 3) many MOF structures contain open metal sites (e.g., Ni(II), Cu(II), Co(II), etc.) that might effectively mitigate the shuttling effect of polysulfides through strong Lewis acid–base interaction to avoid the capacity attenuation as much as possible.^[^ ^30^ ^]^ Up to date, the types of reported MOF‐based Li–S separators can be mainly classified into two forms: MOF‐based composite membranes with polymers or additives as binders^[^ ^31^ ^]^ and MOF film coated commercial separators.^[^ ^32^, ^33^ ^]^ Diverse fabrication methods such as in situ growth of MOF nanocrystals on polypropylene (PP)^[^ ^34^ ^]^ or direct mixing the presynthesized MOFs with additives followed by casting or vacuum filtration have been proposed.^[^ ^35^, ^36^ ^]^ Nevertheless, similar bottlenecks existed for the in situ growth or direct mixing method as mentioned above and all of these methods are hard in mass production, which has largely limited the development of separators for Li–S battery. Therefore, it is demanded to develop novel techniques for the rapid production of MOF‐based MMMs to satisfy the demand of applications like Li–S battery to inhibit polysulfide shuttling and improve the battery performance.^[^ ^37^, ^38^ ^]^

**Figure 1:**
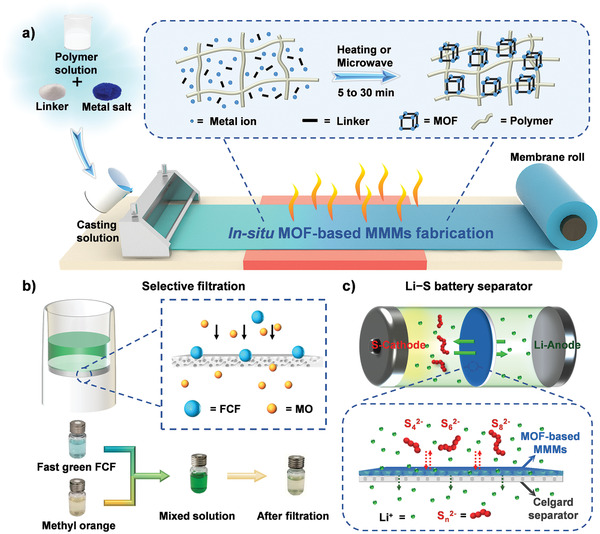
Schematic diagram of in situ heat‐assisted solvent‐evaporation (HASE) method for the fabrication of MOF‐based MMMs and their potential applications in filtration and Li–S battery separator.

To this end, we anticipate to directly mix MOF precursors with polymers in solvent followed by casting and temperature‐controlled heating processes to simultaneously achieve the in situ MOF growth and rapid membrane fabrication (**Figure** **1**). During this process, the well‐controlled temperature might properly adjust the solvent evaporation rate to balance the trade‐off between MOF synthesis and defect generation to produce porous MOF‐based MMMs. As a proof‐of‐concept, we report a facile in situ heat‐assisted solvent‐evaporation (HASE) method to facilely fabricate porous and robust MOF‐based MMMs in industrial‐level efficiency (Figure 1). This method is suitable for various MOFs and polymers with minimum reaction time to be 5 min. The obtained MMMs with high uniformity, excellent robustness, well‐tuned loading and thickness can be massively produced with a production rate of ≈4 m h^−1^ in a batch experiment in lab scale and ≈100 m per day, which is superior to conventional MOF‐based MMMs fabrication methods that might generally take a few days when taking MOF synthesis into consideration. Besides, they exhibit high performances in contaminate removal and Li–S battery. For example, the robust MOF‐based MMMs can serve as powerful separators in Li–S cell and best of them, HPP‐20 (stands for HKUST‐1@PVDF‐HFP‐20@PP) based cell presents a capacity of 1163.7 mAh g^−1^ in the first cycle and 500.7 mAh g^−1^ after 700 cycles at 0.5 C (0.08% fading per cycle), which is superior to most of reported MOF‐based separators. This efficient fabrication method with high production efficiency for MOF‐based MMMs will provide solid basis for the potential industrialization process of MOFs.

## 2 Results and Discussion

The facile in situ HASE method for the fabrication of MOF‐based MMMs can be simply concluded into three steps: mixture solution preparation, casting, and heating treatment (oven‐heating or microwave‐assisted heating). The detail of the HASE method is presented as follows. Certain amount of polymer powder is dissolved in *N*,*N*‐dimethylformamide (DMF) under stirring to form a transparent solution. After that, the linker and metal salt are mixed in DMF solution and stirred at room temperature to achieve homogenous solution. The solution is then casted onto a glass substrate using a doctor blade with defined thickness. Then the glass substrate is heat treated in an oven for 30 min or microwave reactor for 5 min. A stand‐alone MOF@Polymer membrane with well‐defined thickness and MOF loading (MOF@Polymer‐*n* (*n* = 10–70), *n* stands for the different loadings of MOF precursors from 10 to 70 wt%) is obtained after washing with ethanol for three times and drying under vacuum for further characterization (for details see the Experimental Section). Upon tuning the species of MOF precursors and polymers, this method can be extended to various polymers (e.g., polyvinyl chloride (PVC), polyvinylidene fluoride (PVDF), polymethyl methacrylate (PMMA), polystyrene (PS), etc.) and different MOF types (e.g., HKUST‐1 (Hong Kong University of Science and Technology), UiO‐66, NH_2_‐UiO‐66, NENU‐5, etc.). During this heating process, DMF with a boiling point of 153 °C will be readily evaporated with suitable temperature and MOF nanocrystals and giant porosity will be simultaneously produced in the membrane systems, thus achieving the rapid production of MOF‐based porous MMMs.

**Figure 2:**
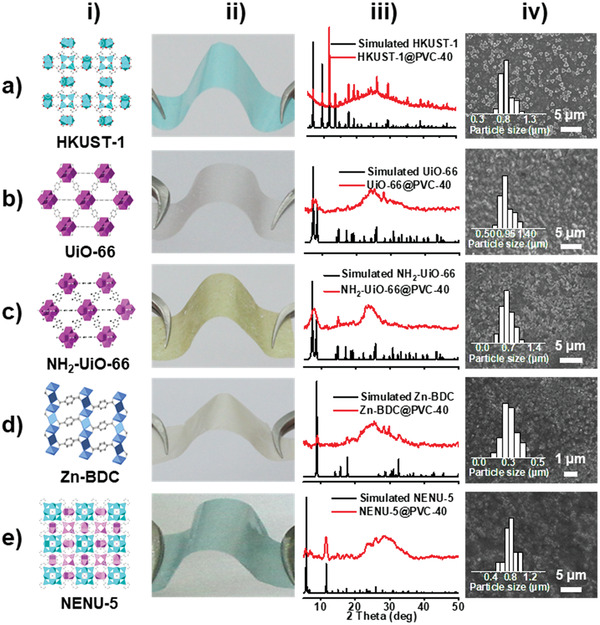
Structure, SEM images, and PXRD patterns of the MOF‐based MMMs (40 wt% loading) obtained from in situ HASE method. a) HKUST‐1@PVC‐40. b) UiO‐66@PVC‐40. c) NH_2_‐UiO‐66@PVC‐40. d) Zn‐Terephthalic acid (BDC)@PVC‐40. e) NENU‐5@ PVC‐40. i) Structure images of MOFs. ii) Photoimages of MOF‐based MMMs. iii) PXRD patterns of MOF‐based MMMs with different structures. iv) SEM images of the prepared MOF‐based MMMs (inset is the particle size distribution calculated based on more than 50 particles).

Taking HKUST‐1@PVC with 40 wt% loading (denoted as HKUST‐1@PVC‐40) for example, the powder X‐ray diffraction pattern (PXRD) shows that HKUST‐1@PVC‐40 displays remained peaks of HKUST‐1 (Figure S1a, Supporting Information). The existence of HKUST‐1 can also be confirmed in the Fourier transform infrared spectroscopy (FT‐IR) spectra (Figure S1b, Supporting Information). To further characterize the morphology, scanning electron microscopy (SEM) test is performed. HKUST‐1 nanoparticles with size of ≈850 nm are uniformly distributed on the membrane (**Figure** **2**). In contrast, the membrane generated from the direct mixing of HKUST‐1 nanoparticles with polymers results in aggregated morphology, which is also clearly visible in the photoimage (Figure S2, Supporting Information). For the MOF‐based MMMs, the more uniform of the nanoparticle distribution, the higher mechanical strength might be achieved. To prove it, the tensile stress test is further conducted and HKUST‐1@PVC‐40 shows higher tensile stress than presynthesized HKUST‐1 based membrane with similar thickness (Figure S3, Supporting Information).

Besides, the thickness and MOF loading of these membranes can be easily tuned. Through controlling the setting thickness of casting blade (from 200 to 1000 µm), the thickness of HKUST‐1@PVC‐40 membrane can be accordingly adjusted (from ≈40 to ≈80 µm) (Table S1, Supporting Information). PXRD tests indicate the remained crystalline phase of HKUST‐1 in the HKUST‐1@PVC‐40 membrane with different thicknesses (Figure S4, Supporting Information). Further revealed by the SEM tests, all of the HKUST‐1 nanoparticles are uniformly distributed on the membranes (Figure S5, Supporting Information). The sizes of HKUST‐1 nanoparticles are measured with a nanomeasurer software and the results show that the sizes of HKUST‐1 nanoparticles become larger with the increase of thickness (Table S1, Supporting Information). This might be ascribed to the longer solvent‐evaporation time for thicker membrane that can guarantee sufficient time for the growth of MOF nanocrystals. Besides, membranes fabricated under different temperatures are also investigated and the sizes of nanoparticles might be smaller and smaller with the increase of temperature. As a proof‐of‐concept, the sizes of HKUST‐1 nanoparticles become smaller when the temperatures are tuned from 80 to 140 °C (e.g., 80 °C, ≈1.48 µm; 100 °C, ≈1.02 µm; 120 °C, ≈850 nm, and 140 °C, ≈200 nm) as proved by the PXRD and SEM tests (Figures S6 and S7, Supporting Information). Furthermore, different MOF loadings can be easily controlled through the adjustment of concentrations for MOF precursor solution. Specially, the loadings of MOF can be well‐tuned from about 10 to 40 wt% with intact topology of MOF nanocrystals in the membranes supported by PXRD and FT‐IR tests (Figure S8, Supporting Information). Moreover, the density of MOF particles gradually becomes denser with the increase of MOF loading as presented in the SEM tests (Figure S9, Supporting Information). Besides, the tensile stress tests show that the stress of the membrane decreases with the increase of the MOF loading (Figure S10, Supporting Information). Besides, positronium annihilation lifetime spectroscopy has been applied to measure the free volume in the fabricated MMM. Take HKUST‐1@PVDF‐40 as an example, the average free void size of HKUST‐1@PVDF‐40 is calculated to be ≈2.9 Å with a free volume of ≈108.961 Å^3^ (for details see the Experimental Section).

In addition, this powerful method can be extended to various MOF systems and polymers. Except for HKUST‐1, different MOF systems including UiO‐66, NH_2_‐UiO‐66, Zn‐MOF, and NENU‐5 are explored. Conducted under similar procedures as that of HKUST‐1@PVC‐40, various MOF@PVC‐40 membranes with uniformly distributed morphology are obtained as supported by PXRD and SEM tests (Figure 2). Tensile stress tests are performed to show the robustness of these membranes and various stress are achieved for UiO‐66@PVC‐40 (≈6.7 MPa, ≈3.1%), NH_2_‐UiO‐66@PVC‐40 (≈2.8 MPa, ≈1.1%), Zn‐BDC@PVC‐40 (≈3.7 MPa, ≈1.5%), and NENU‐5@PVC‐40 (≈1.3 MPa, ≈0.59%), respectively (Figure S11, Supporting Information). Besides, various polymers (i.e., PVDF, poly(vinylidene fluoride‐*co*‐hexa‐fluoropropylene) (PVDF‐HFP) PMMA and PS) are also investigated. Performed under similar processes, HKUST‐1@PVDF‐40, HKUST‐1@PVDF‐HFP‐40, HKUST‐1@PS‐40, and HKUST‐1@PMMA‐40 are prepared verified by PXRD and SEM tests (Figure S12, Supporting Information). Taking PVC and PVDF based membranes as examples, HKUST‐1 nanoparticles are uniformly distributed on HKUST‐1@PVDF‐40 or HKUST‐1@PVC‐40 compared with the flat and clean surface of only polymer‐based membrane (Figure 2 and Figures S12 and S13, Supporting Information). To prove the generality of this method, PVDF can also be applied to combine various MOFs to generate diverse MOF‐based MMMs (Figure S14, Supporting Information). Besides, tensile stress tests show high stress are achieved for HKUST‐1@PVDF‐40 (≈13 MPa, ≈2.5%), HKUST‐1@PS‐40 (≈18 MPa, ≈2.6%), and HKUST‐1@PMMA‐40 (≈12 MPa, ≈2.6%), respectively (Figure S15, Supporting Information). All of the mechanical stresses of these MOF‐based MMMs through such HASE method are higher than those MMMs prepared from presynthesis method, which further certifies the superiority of this method (Figure S16, Supporting Information).

The versatility of this method holds great promise in practical applications yet it is still highly demanded to reduce the membrane fabrication time (≈30 min) to shorter ones that can largely mitigate the energy and time consumption. Microwave‐assisted method, which has been intensively studied in the hydrothermal syntheses of MOF‐based nanocrystals or nanocomposites, might be an alternative choice.^[^ ^39^ ^]^ Despite some pioneering work of microwave‐assisted method in the fabrication of MOF‐based crystalline film,^[^ ^40^ ^]^ the utilization of this method has been rarely investigated in the fabrication of MOF‐based MMMs. Interestingly, these robust membranes can be readily produced under microwave‐assisted heating conditions. Taking HKUST‐1@PVC‐40 for example, it can be obtained in just 5 min compared with that of oven‐heating method (≈30 min) as proved by PXRD test (Figure S17a, Supporting Information). SEM test indicates that HKUST‐1 nanocrystals with sizes about 270 nm are uniformly distributed on the membrane, which is much smaller than that of synthesized from oven‐heating method (≈850 nm) (Figure S17a, Supporting Information). This might be ascribed to the higher energy and shorter reaction time of the microwave‐assisted method. Except for HKUST‐1, representative MOF systems such as UiO‐66, NH_2_‐UiO‐66, and NENU‐5 can also be fabricated into membranes. The successful preparation of UiO‐66@PVC‐40, NH_2_‐UiO‐66@PVC‐40 and NENU‐5@PVC‐40 are proved by PXRD tests, in which the crystalline phases of the membranes match well with the simulated MOFs (Figure S17, Supporting Information). SEM tests show that these membranes display similar uniformly distributed morphology as that of membranes produced through oven‐heating method except that the sizes of MOF nanoparticle are smaller (Table S2, Supporting Information). Furthermore, the microwave‐assisted method inherits the versatility of oven‐heating method and can be applied into various polymer systems. For example, the fabrication of UiO‐66@PVDF‐40, NH_2_‐UiO‐66@PVDF‐40, and NENU‐5@PVDF‐40 can also be achieved in just 5 min as verified by PXRD and SEM tests (Figure S18, Supporting Information). This powerful method coupled with facile microwave‐assisted technique might extend it into industrial‐scale production to meet the practical demand.

Nowadays, MOF‐based MMMs especially for those fabricated through in situ methods,^[^ ^41^, ^42^, ^43^, ^44^, ^45^ ^]^ the loadings of MOFs are mostly below 40 wt% owing to their low precursor transformation efficiency under large amount of solvent system.^[^ ^46^ ^]^ Obtained from such a facile in situ HASE method with only small amount of solvent, the precursors might be efficiently and readily transformed into MOF nanocrystals under heating or microwave‐assisted conditions to achieve higher loadings. To this end, MOF membranes with high loadings are explored with this method. UiO‐66@PVDF is selected as a model membrane to investigate. UiO‐66@PVDF‐50, UiO‐66@PVDF‐60 and UiO‐66@PVDF‐70 can be successfully fabricated as proved by PXRD and SEM tests through oven‐heating method (Figures S19–S21, Supporting Information). The robustness of the membrane material is one of the key parameters to be evaluated especially for membrane with high MOF loadings. Specially, high‐loading MOF‐based MMMs like UiO‐66@PVDF‐70 displays remained high tensile stress as verified by the preliminary tensile test with a piece of membrane (width, 1.5 cm) holding a weight (≈0.6 kg) downside (Figure S21e, Supporting Information). Furthermore, tensile stress tests indicate that these high MOF loading membranes exhibit slightly lower tensile stress compared with that of UiO‐66@PVDF‐40 (Figure S21f, Supporting Information). Besides, microwave‐assisted method can also be applied in the production of MOF‐based MMMs with high loadings and for example, UiO‐66@PVDF‐70 can be successfully produced as proved by the PXRD and SEM tests (Figure S22, Supporting Information).

**Figure 3:**
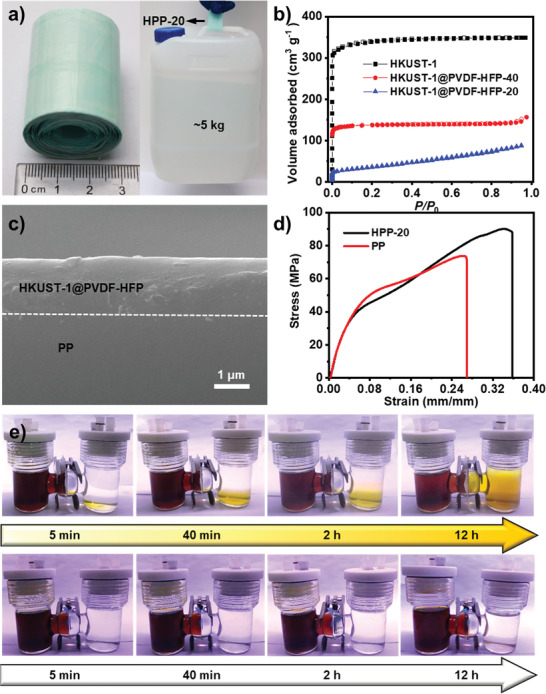
Characterization and polysulfide permeation tests of HPP‐20 separator. a) Photoimages of HPP‐20 separator obtained in large quantities (≈4 m in a batch experiment in lab scale, width, ≈4 cm) and tensile stress test (≈5 kg bucket holding downside). b) N_2_ sorption curves. c) SEM image of the cross‐section for HPP‐20, the thickness is about 1.5 µm. d) Stress–strain curve of HPP‐20 and PP. e) Polysulfides permeation tests for the PP and HPP‐20.

For most of MOF‐based MMMs, the maintenance of MOF porosity remains a great challenge yet still lack alternative methods. Despite some presynthesis methods (e.g., casting,^[^ ^47^, ^48^ ^]^ photoinduced presynthetic polymerization^[^ ^49^ ^]^ or thermally induced phase separation‐hot pressing strategy)^[^ ^46^ ^]^ reported remained porosity of MOFs in the MMMs, the presynthesis methods would still face drawbacks such as agglomeration of nanoparticles, excessive energy consumption during MOF synthesis, and activation. Fabricated through the in situ HASE method, in which the solvent can be readily evaporated during the process to create large number of defects, thus‐obtained membranes might maintain most of the MOF porosity. As a proof‐of‐concept, HKUST‐1@PVDF‐HFP‐40 exhibits an *S*_BET_ of 487 m^2^ g^−1^ through Brunner–Emmet–Teller (BET) measurements, which accords with that of pristine HKUST‐1 (1187 m^2^ g^−1^) when taking 40 wt% loading into consideration (**Figure** **3**b). HKUST‐1@PVDF‐HFP‐40 displays a pore size distribution centered at 0.89 nm, close to that of HKUST‐1 (0.95 nm), which implies the accessible porosity of HKUST‐1 in the membrane. When the loading is 20 wt%, the *S*_BET_ is calculated to be 99 m^2^ g^−1^, which is slightly lower than the target one (237 m^2^ g^−1^) (Figure 3b). This might be attributed to be partially blocked effect of polymer, which is also proved by the pore size distribution that two kinds of pores centered at 0.75 and 0.81 nm are detected (Figure S23, Supporting Information).

The industrial‐level efficiency and versatile in situ HASE method endows these membranes with tremendous porosity, high mechanical strength and uniformly distributed nanoparticles that might render them as promising candidates in separation. Especially for the removal of low‐concentration contaminant, it has remained a giant challenge for most of filters. Based on this and in order to explore the potential applications of the membranes, UiO‐66@PVDF‐60 is applied as the separator for dye separation experiments both for one and more dye components. The chemical stability of UiO‐66@PVDF‐60 has been conducted through immersing the membrane in HCl (pH = 1) and NaOH solution (pH = 10) for 3 d. The results show that the PXRD patterns of the samples remain almost unchanged when compared with prepared ones (Figure S25, Supporting Information). For one dye component removal, Fast Green FCF (FCF) solution (12 × 10^−6^ m) is filtrated through a filter device (membrane inside, ≈2 cm in diameter) with a rate of ≈1 mL s^−1^ (Figure S24a, Supporting Information). After filtration, the color of the solution becomes transparent and the eluate is analyzed by UV/Vis spectroscopy (*I* = 624.5 nm). Remarkably, 98.2 ± 2.0% removal efficiency is detected in the filtrated FCF solution (Figure S26, Supporting Information). We have studied the cycle performance of the membrane and the removal efficiency can still maintain up to 93.2% ± 1.9% after five filtration cycles (Figure S26, Supporting Information). In fact, most of contaminates are in mixed‐form in natural environment and might pose higher challenge for the filtration techniques. In this work, two kinds of dye molecules (i.e., FCF and methyl orange (MO)) in mixed‐form are regard as the stimulants in natural environment. Typically, the same volume of 12 × 10^−6^ m FCF and 30 × 10^−6^ m MO solution are mixed and filtrated through UiO‐66@PVDF‐60. Interestingly 97.6% ± 2.4% of the FCF solution is blocked and 98.0 ± 2.0% of MO solution is passed (Figure S24b, Supporting Information). The high contaminant separation efficiency indicates the size selectivity ability of the obtained membranes, which also implies them to be promising candidates for more applications like the separators in Li–S battery.

Produced through this powerful method, the obtained membranes with remained porosity, high robustness and size selectivity ability might be promising alternatives as separators in Li–S cell. To investigate it, HKUST‐1@PVDF‐HFP is selected as the desired example owing to HKUST‐1 and PVDF‐HFP have been studied in the separator of Li–S cell.^[^ ^50^, ^51^ ^]^ Based on the HASE method, HKUST‐1@PVDF‐HFP membranes with different thicknesses (10–75 µm) and loadings (precursor loading from 10 to 40 wt%, denoted as HPP‐10–40) are casted onto commercial PP using a doctor blade. After heat treatment, HKUST‐1@PVDF‐HFP can be tightly coated onto PP. Taking HKUST‐1@PVDF‐HFP‐20@PP (denoted as HPP‐20) for example, the membrane is successfully fabricated as proved by PXRD tests (Figure S27, Supporting Information). SEM images of HPP‐20 and PP show that the macropores (≈300 nm) in PP are fully covered by HKUST‐1@PVDF‐HFP‐20 (Figure S28, Supporting Information). The cross‐section image of HPP‐20 presents that the thickness of coated membrane is about 1.5 µm (Figure 3c). As mentioned above, HKUST‐1@PVDF‐HFP‐20 has an *S*_BET_ of 99 m^2^ g^−1^ and displays pore size distribution centered at 0.75 and 0.81 nm (Figure S23, Supporting Information). The pores in the membrane are fully accessible yet they are still slightly larger than the sizes of polysulfide (0.51–0.68 nm).^[^ ^52^ ^]^ Interestingly, polysulfide permeation test shows that HPP‐20 can efficiently restrain the shuttling of polysulfides for more than 12 h (Figure 3e). This result is far superior to PP, in which the permeation phenomenon occurs after only 5 min and the color of right side solution changes to brown after 12 h (Figure 3e). The high restraining efficiency might be attributed to the porosity effect^[^ ^53^ ^]^ and ample open Cu metal sites in HPP‐20 that might join together to minimize the shutting effect of polysulfides.^[^ ^54^ ^]^ Notably, HPP‐20 is ease of mass production and ≈4 m HPP‐20 can be readily produced in a batch experiment in lab scale (Figure 3a). Noteworthy, it is found that the stress of HPP‐20 can be largely enhanced and reaches to ≈89 MPa, which is much larger than PP (≈74 MPa) and pure PVDF‐HFP (≈29.9 MPa) (Figure 3d and Figure S29, Supporting Information). This result is also visibly proved by the tensile test with a piece of HPP‐20 (width, ≈4.0 cm) holding a ≈5 kg bucket downside (Figure 3a).

**Figure 4:**
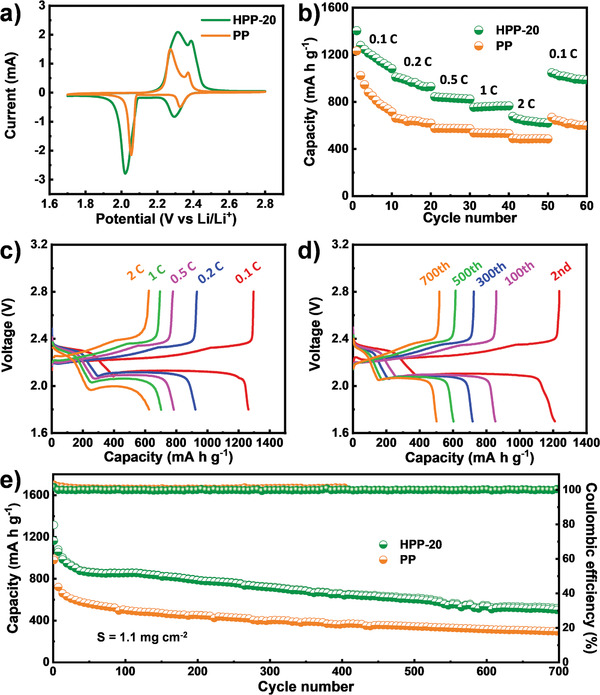
The cell performances of HPP‐20 and PP based Li–S cells. a) CV curves tested at a scan rate of 0.1 mV s^−1^. b) Rate capabilities of battery using different separators under the rate of 0.1, 0.2, 0.5, 1, and 2 C. c) Galvanostatic charge–discharge profiles tested at the rate of 0.1, 0.2, 0.5, 1, and 2 C. d) Charge/discharge curves in cycle test for HPP‐20 based Li–S cell performed at 0.5 C. e) Long life cycle tests for HPP‐20 and PP based Li–S cells measured at 0.5 C.

The combination of the possibility in mass production, high mechanical strength, and restraining efficiency for polysulfides in HPP‐20 makes it to be potential candidate as the separator in Li–S battery. To test it, HPP‐20 serving as the separator are assembled in coin cell with Ketjen black (KB)/S (S content of 70 wt%) as cathode materials and Li metal foils as anodes. To investigate the cell performance, regular electrochemical tests, such as galvanostatic discharge/charge, cyclic voltammetry (CV), and electrochemical impedance spectra (EIS) have been conducted.^[^ ^55^ ^]^ For comparison, the cell performances of pure PP based Li–S cell are also tested. CV tests are performed to study the conversion of polysulfides in the cell. The CV curves of HPP‐20 based Li–S cell exhibits two reduction peaks centered at 2.3 and 2.0 V, representing the reduction of elemental sulfur to soluble high order polysulfides (Li_2_S*_n_*, 4 < *n* < 8) and their further reduction to solid lithium sulfides (Li_2_S_2_/Li_2_S), respectively. In the anodic scans, overlapped peaks fitted around 2.4 V are assigned to the oxidation of Li_2_S/Li_2_S_2_ to Li_2_S_8_/S_8_ (**Figure** **4**a).

Furthermore, the rate performance of the cell is evaluated by galvanostatic charge/discharge at various rates from 0.1 to 2 C within a potential window of 1.7–2.8 V (Figure 4b). It can be seen that the HPP‐20 based Li–S cell shows an initial capacity of 1404.6 mAh g^−1^ at 0.1 C, which is 171.1 mAh g^−1^ larger than that of PP based Li–S cell. The additional capacity for HPP‐20 based Li–S cell might be attributed to the efficient inhibition of polysulfides shuttling, leading to a high utilization of active materials. For the capacity, HPP‐20 based Li–S cell delivers high capacity of 1010.5, 843.7, 753.5, and 676.0 mAh g^−1^ at the rates of 0.2, 0.5, 1, and 2 C, respectively (Figure 4a). The performance is superior to PP based Li–S cell, in which the capacity decreases rapidly with the increase of current density (i.e., 662.2, 574.5, 536.5, and 488.1 mAh g^−1^ at the rates of 0.2, 0.5, 1, and 2 C, respectively) (Figure 4b). Besides, all the discharge curves at various rates for the cell exhibit two typical discharge plateaus, relating to two typical reduction stages of Li–S cell (Figure 4c), which agree with the CV curves (Figure S30, Supporting Information). Finally, the capacity returns to 1046.8 mAh g^−1^ at 0.1 C, suggesting the good rate performance of the cell. The improved rate performance of HPP‐20 based Li–S cell might be ascribed to two possible factors: 1) the highly ordered pore structures of MOFs are conducive to the transport of Li^+^ and 2) the effective inhibition of polysulfides shuttling can largely improve the reutilization of active materials.

The cycle stability is vital for the long‐term application of Li–S battery. The cycling tests for different separators are studied by galvanostatic charge/discharge test at 0.5 C. The initial capacity is measured to be 1163.7 mAh g^−1^ and the capacity in the 100th, 300th, 500th, and 700th cycles are 853.7, 717.2, 602.6, and 500.7 mAh g^−1^, respectively, proven beneficial retention of capacity (43.0% retention, 0.08% fading per cycle for 700th cycle) (Figure 4d). To the best of our knowledge, the performance is superior to most of reported MOF‐based separators (Table S3, Supporting Information). As comparison, PP based Li–S cell only exhibits an initial capacity of 979.4 mAh g^−1^. Besides, the capacity decreases rapidly as the cell charging/discharging constantly, especially in the first 40 cycles (Figure 4d). After 700 cycles, it only can deliver a low capacity of 296.3 mAh g^−1^ with a very low capacity retention (30.2%) (Figure 4e). Besides, the EIS of the cells has been tested for both Li–S cells with different separator systems and present two similar Nyquist plots. Semicircles in the high‐medium frequency region are ascribed to the charge‐transfer resistance (*R*_ct_) and straight lines in the low frequency region are corresponded to a mass‐transfer process. It can be seen that the *R*_ct_ value of the cell with addition of HPP‐20 (5.55 Ω) is lower than that of the cell with pure PP (6.69 Ω), implying a much more rapid electron transfer than PP (Figure S31, Supporting Information).

For comparison, HKUST‐1@PVDF‐HFP with different thicknesses (10–75 µm) and loadings (10–40 wt%) are coated onto PP to investigate their cycling performances by galvanostatic charge/discharge test at 0.5 C. For the coatings with thicknesses about 10, 50, and 75 µm, the corresponding Li–S cells present initial capacity of 1062.8, 775.7, and 181.7 mAh g^−1^, and after 150 cycles, the capacity of them fade to 618.7, 357.8, and 132.7 mAh g^−1^, respectively (Figure S32a, Supporting Information). The performances of these cells are much lower than that of separator with 25 µm coating as presented above. The reason for the drastically decrease in capacity may be that the thinner the thickness, the lower the barrier properties of the material for polysulfides. What is more, the increase in thickness might induce the decrease in wettability of the separator with the electrolyte. Similarly, HKUST‐1@PVDF‐HFP with different MOF loadings (i.e., 10, 30, and 40 wt%) are casted under similar thickness onto PP (denoted as HPP‐10, HPP‐30 and HPP‐40) to investigate their cell performances. The initial capacity of HPP‐10, HPP‐30, and HPP‐40 are 921.6, 942.6, and 718.1 mAh g^−1^, and the capacity are decreased to 516, 373, and 368 mAh g^−1^ after 150 cycles, respectively (Figure S32b, Supporting Information). Besides, different kinds of MOF‐based MMMs (i.e., UiO‐66@PVDF‐HFP and NH_2_‐UiO‐66@PVDF‐HFP) are successfully coated onto PP and applied as Li–S cell separators (Figure S33, Supporting Information). UiO‐66 and NH_2_‐UiO‐66 based Li–S cell separators are denoted as UPP‐20 and NPP‐20, respectively. The initial capacity of UPP‐20 and NPP‐20 are 1071.0 and 1207.1 mAh g^−1^ and their capacity fade to 658.6 and 750.1 mAh g^−1^ after 150 cycles, respectively (Figure S34, Supporting Information).

The PXRD pattern shows that the inert structure of HPP‐20 remains stable after cell test (Figure S35, Supporting Information). FT‐IR spectra of the HPP‐20 after test displays a peak at 613 cm^−1^, which is ascribed to the generation of Cu‐S bond and further proves the adsorption effect of S on the ample open Cu sites in the membrane (Figure S36, Supporting Information). Further certified by the SEM test, the morphology of the HPP‐20 in both top‐view and cross‐section images after cell test remains almost unchanged compared with the state before test, implying the high durability of HPP‐20 during cell tests (Figure 3c and Figures S35 and S36, Supporting Information).

## 3 Conclusion

In conclusion, an in situ HASE method for rapid production of MOF‐based porous MMMs in industrial‐level efficiency has been proposed. It can be extended to various polymers (i.e., PVC, PVDF, PMMA, PS, etc.) and different MOF types (i.e., HKUST‐1, UiO‐66, NH_2_‐UiO‐66, etc.). Noteworthy, the production efficiency of this one pot in situ method can be as less as 5 min with very little solvent utilized and can continuously and massively produce membranes in industrial‐level efficiency (≈4 m in a batch experiment in lab scale). The powerful method endows thus‐obtained MMMs with high mechanical strength and accessible pores, which can be utilized in high‐efficiency (>98%) dye separation and selective filtration of mixed components. Furthermore, the MOF‐based MMMs produced through such a facile and scale method can also serve as powerful coatings to decorate commercial PP with largely improved robustness. The decorated PP can be readily assembled as separator in Li–S battery and exhibits far superior performance to commercial PP. Best of them, HPP‐20 based Li–S cell exhibits a capacity of 1163.7 mAh g^−1^ in the first cycle and a capacity retention of 500.7 mAh g^−1^ after 700 cycles at 0.5 C with a low capacity decay rate (0.08% fading per cycle), which is superior to most of reported MOF‐based separators. The powerful method that can facilely and massively produce MOF‐based porous MMMs applicable in contaminant removal and energy storage applications might largely facilitate the industrialization process of MOFs.

## 4 Experimental Section

##### Fabrication of HKUST‐1@Polymer‐40 through Oven‐Heating Method

Taking HKUST‐1@PVC‐40 for example, PVC (1.4 g) was dissolved in DMF (10 mL) under stirring. Cu(NO_3_)_2_·3H_2_O (2.0 mL, 1.0 mmol mL^−1^) and H_3_BTC (8.0 mL, 0.26 mmol mL^−1^) in DMF solution were mixed and added into the polymer solution under stirring. Then, a certain amount of solution was casted onto glass substrate with defined thickness with a doctor blade (device see Figure S39, Supporting Information). After that, the glass substrate was transferred to an oven and heated at 120 °C for 30 min. After cooling to room temperature, the membrane was peeled off from the substrate followed with soaking in ethanol for several times and drying at 60 °C under vacuum to obtain HKUST‐1@PVC‐40. For the fabrication of HKUST‐1@Polymer with other polymer types (i.e., PS, PMMA, and PVDF), the procedures were similar as that of HKUST‐1@PVC‐40 except different polymers were used. To achieve HKUST‐1@PVC with different loadings, the molar ratio of the precursors changed accordingly with the target loading and the procedures were the same as HKUST‐1@PVC‐40.

##### Fabrication of NENU‐5@Polymer‐40 through Oven‐Heating Method

For the fabrication of NENU‐5@Polymer‐40 (i.e., PVC and PVDF), the procedures were similar as that of HKUST‐1@PVC‐40 except different MOF precursors and temperature are used. The preparation of MOF precursors was as follows. Cu(CH_3_COO)_2_·H_2_O (1.7 mmol) and H_3_[P(Mo_3_O_10_)_4_] (0.15 mmol) were mixed in 5.0 mL DMF and added into the polymer solution under stirring. After that H_3_BTC DMF solution (5.0 mL, 0.29 mmol mL^−1^) was added followed by similar casting, oven‐heating (temperature, 100 °C), and post‐treatment processes.

##### Fabrication of UiO‐66@Polymer‐40 through Oven‐Heating Method

For the fabrication of UiO‐66@Polymer‐40 (i.e., PVC and PVDF), the procedures were similar as that of HKUST‐1@PVC‐40 except different MOF precursors were used. The preparation of MOF precursors was as follows. ZrCl_4_ DMF solution (2.0 mL, 1.17 mmol mL^−1^) and 1,4‐dicarboxybenzene in DMF solutions (8.0 mL, 0.29 mmol mL^−1^) were mixed in the solution followed by similar casting, oven‐heating (temperature, 120 °C), and post‐treatment processes.

##### Fabrication of NH_2_‐UiO‐66@Polymer‐40 through Oven‐Heating Method

For the fabrication of NH_2_‐UiO‐66@Polymer‐40 (i.e., PVC and PVDF), the procedures were similar as that of HKUST‐1@PVC‐40 except different MOF precursors were used. The preparation of MOF precursors was as follows. ZrCl_4_ DMF solution (2.0 mL, 1.17 mmol mL^−1^) and 2‐aminoterephthalic acid in DMF solutions (8.0 mL, 0.28 mmol mL^−1^) were mixed in the solution followed by similar casting, oven‐heating (temperature, 120 °C), and post‐treatment processes.

##### Fabrication of Zn‐BDC@Polymer‐40 through Oven‐Heating Method

For the fabrication of Zn‐BDC@Polymer‐40 (i.e., PVC and PVDF), the procedures were similar as that of HKUST‐1@PVC‐40 except different MOF precursors were used. The preparation of MOF precursors was as follows. Zn(NO_3_)_2_·6H_2_O (5.0 mL, 0.53 mmol mL^−1^) and 1,4‐dicarboxybenzene in DMF solutions (5.0 mL, 0.18 mmol mL^−1^) were mixed followed by similar casting, oven‐heating (temperature, 120 °C), and post‐treatment processes.

##### Fabrication of MOP‐1@PVDF‐40 through Oven‐Heating Method

For the fabrication of metal‐organic polyhedra (MOP)‐1@PVDF‐40, the procedures were similar as that of HKUST‐1@PVC‐40 except different MOF precursors were used. The preparation of MOF precursors was as follows. Cu(NO_3_)_2_·3H_2_O (5.0 mL, 0.49 mmol mL^−1^) and isophthalic acid in DMF solutions (5.0 mL, 0.39 mmol mL^−1^) were mixed followed by similar casting, oven‐heating (temperature, 120 °C), and post‐treatment processes.

##### Fabrication of MOF@Polymer‐40 through Microwave‐Assisted Method

For the fabrication of MOFs (e.g., HKUST‐1, NENU‐5, UiO‐66, and NH_2_‐UiO‐66) based MMMs with different polymers (i.e., PVC and PVDF) through microwave‐assisted method, the casting and post‐treatment procedures were similar as that of oven‐heating method except the heating processes were conducted in microwave reactor (high power, 5 min).

##### Fabrication of HPP‐*n* (*n* = 10, 20, 30, 40)

Taking HPP‐20 as an example, PVC (1.4 g) was dissolved in DMF (5.0 mL) under stirring. Cu(NO_3_)_2_·3H_2_O (1.0 mL, 0.80 mmol mL^−1^), and H_3_BTC (4.0 mL, 0.18 mmol mL^−1^) in DMF solutions were mixed and added into the polymer solution under stirring. Then, a certain amount of solution was casted onto PP with defined thickness with a doctor blade. After that, the glass substrate was transferred to an oven and heat at 120 °C for 30 min. After cooling to room temperature, HPP‐20 was peeled off from the substrate followed with soaking in ethanol for several times and drying at 60 °C under vacuum. To achieve HPP‐*n* (*n* = 10, 30, 40), the molar ratio of the precursors changes accordingly with the target loading and the procedures are the same as HPP‐20.

##### Synthesis of Li_2_S_6_ in DOL and DME

The synthesis of Li_2_S_6_ electrolyte followed previously reported method.^[^ ^28^ ^]^ Li_2_S and S were mixed with a molar ratio of 4:3 in a glass bottle (20 mL) under the protection of Ar. Then a mixed solvent of 1,3‐dioxolane (DOL) and 1,2‐dimethoxyethane (DME) (v: v = 1:1) was introduced in the glass bottle followed with heating at 50 °C for 12 h to make Li_2_S and S_8_ react completely.

##### Tensile Test

The obtained membrane was cut into a strip sample (length, ≈5 cm and width, ≈15 mm). Before the test, the clamp spacing was controlled to be 2–3 cm. The stretching rate was controlled at 5 mm min^−1^ and the timely data were recorded with a computer (Figure S40, Supporting Information).

##### Li–S Coin Cell Assembly

All Li–S coin cells were assembled in an Ar filled glove box with moisture and oxygen contents below 1 ppm. The 2032 coin cell was used to assemble cell for the electrochemical test of Li–S battery. The cathode materials were prepared by mixing KB@S (KB: S = 3:7), the sulfur content in the composite was measured to be 69.1 wt% by thermogravimetric analyzer (Figure S41, Supporting Information). KB@S, Super P (SP), and PVDF (weight ratio, KB@S: SP: PVDF = 7:2:1) were mixed in *N*‐methyl‐2‐pyrrolidinone (>99.9%, Sigma‐Aldrich). The obtained slurry was coated onto acetylene black decorated Al foil. After drying at 80 °C for 10 h under vacuum, the mixture coated Al foil was cut into wafers with a diameter of 14 mm and used as cathodes (mass loading of sulfur, 0.8–1.2 mg cm^−2^). Li metal foil in round shape (diameter, 14 mm) was used as the anode material. The electrolyte was 1 m LiTFSI and 2 wt% LiNO_3_ in DOL and DME (volume ratio, 1:1). The addition amount of electrolyte in every cell was about 40 µL. The commercial PP and HPP‐20 with diameter of 18 mm were used as the separators. The galvanostatic charge/discharge tests were carried out on a Land Battery Measurement System (Land, CT2001A, China) at room temperature.

## Conflict of Interest

The authors declare no conflict of interest.

## Supporting information

Supporting Information
