# Supplementary material for: Rapid Production of Metal–Organic Frameworks Based Separators in Industrial‐Level Efficiency
Source: Adv Sci (Weinh). 2020 Nov 6;7(24):2002190. doi: 10.1002/advs.202002190 (PMC7740102; doi:10.1002/advs.202002190)
Supplement: Supplementary file 1 — Supporting Information [file ADVS-7-2002190-s001.pdf]

© 2020 Wiley-VCH GmbH

ADVANCED HEALTHCARE MATERIALS

Supporting Information

for *Adv. Healthcare Mater.*, DOI: 10.1002/advs.202002190

Rapid Production of Metal-Organic Frameworks based Separators in Industrial-level Efficiency

Guang-Kuo Gao, Yi-Rong Wang, Hong-Jing Zhu, Yifa Chen, Ru-Xin Yang, Cheng Jiang, Huiyuan Ma,* and Ya-Qian Lan,**

Materials and methods

Materials: All solvents and reagents obtained from commercial sources are used without further purification. $Cu(NO_3)_2·3H_2O$ (99.5%) and $Cu(CH_3COO)_2·H_2O$ (AR) are obtained from Sinopharm Chemical Reagent Co., Ltd. 1,3,5-Benzenetricarboxylic acid (H_3BTC) (98%), $Zn(NO_3)_2·6H_2O$ (AR), poly(methyl methacrylate) (PMMA, average M_w ~550,000) and sulfur powder (~325 mesh, 95%) are purchased from Alfa Aesar. $ZrCl_4$ (98%) is purchased from Acros Chemicals. Isophthalic acid (AR), polyvinylidene fluoride (PVDF, melt viscosity (230°, 100 s^{-1}) 23500-29500 poise) and polyvinyl chloride (PVC, K-value 59-55) are purchased from MACKLIN reagent. $H_3[P(Mo_3O_{10})_4]$ (AR), 2-aminoterephthalic acid (> 98.0%) and 1,4-dicarboxybenzene (99%) are purchased from Aladdin reagent. Methyl orange (98.0%) and Fast Green FCF (> 85.0%) are obtained from TCI. Polystyrene (PS, average M_w ~192,000) and poly(vinylidene fluoride-co-hexa-fluoropropylene) (PVDF-HFP, average M_n ~110,000) are purchased from Aldrich. Celgard 2400 (PP), Ketjen black (KB) and Super P are obtained from commercial sources.

Characterizations and instruments: Field emission scanning electron microscopy (SEM, Quanta 250F) equipped with an energy dispersive X-ray spectrometer (EDS) is applied to investigate the morphology and composition information of the samples. Powder X-Ray diffraction (PXRD) patterns of samples are carried out on a D/max 2500 VL/PC diffractometer (Japan) equipped with Cu Kα radiation (λ = 1.54060 Å). FT-IR spectra are recorded on Bruker (ALPHA) spectrometer. N_2 isotherm is measured using a Bruker D8 Advance automatic volumetric gas adsorption analyzer. The model of tensile test instrument is G425 Instron universal test machine. Electrochemical impedance spectroscopy (EIS) measurements and cyclic voltammetry (CV) are conducted on CHI 660D (Shanghai, China) electrochemical workstation at room temperature. Thermo gravimetric analyzer (TGA) is carried out under nitrogen with a Perkin-Elmer TG-7 analyzer heated from room temperature to 500 °C at a ramp rate 5 °C min^{-1}. Microwave oven (Midea, MM721NG1-PS, 700 W) is used as the microwave reactor for the fabrication of membranes.

The source of positrons was provided by ^{22}Na and the membranes applied in the PALS experiments were cut into round pellet (d, ~1.2 cm) and were stacked on the top of each other (thickness, ~1 mm) to stop ~99% of the incident positrons. The lifetime measurements were tested at 25 ^oC with at least 2 million counts. The lifetime spectra were analyzed with LT 9.0 and a four-finite lifetime component analysis yielded the optimum fit to the experimental data. The average molecular hole size was calculated via the following semi-empirical equation:

$$\tau_j = 0.5\,ns\left[1 - \frac{R_j}{R_j + \Delta R} + \frac{1}{2\pi} sin\left(\frac{2\pi R_j}{R_j + \Delta R}\right)\right]^{-1}$$

where τ_j is the lifetime of *o*-Ps (ns, j = 3 or 4), and 0.5 ns is the spin-averaged lifetime of the *o*-Ps, R_j is the average hole radius (Å), Δ*R* is a fitted empirical electron layer thickness (Δ*R* = 1.66 Å) determined by fitting well-known cavities.

Synthesis of HKUST-1: H_3BTC (0.38 g, 1.8 mmol) is dissolved in DMF (15 mL). $Cu(CH_3COO)_2 \cdot H_2O$ (0.65 g, 3.2 mmol) is dissolved in DMF (15 mL). Then, they are separately added into 150 mL mixed solution with DMF, ethanol and water (volume ratio, 1 : 5 : 5) followed by continuously stirring at room temperature for 3 h. After centrifugation, the obtained sample is washed with DMF and ethanol each for three times. After drying at 80 ^{o}C under vacuum, the powder is collected for further characterization.

Synthesis of UiO-66: The synthesis of UiO-66 follows reported method.[1] Zirconium (IV) chloride ($ZrCl_4$) (61 mg, 0.26 mmol) and terephthalic acid (43 mg, 0.26 mmol) are dissolved in DMF (15 mL) with glacial acetic acid (0.45 mL) in a 20 mL vial. The capped vial is placed in an oven and heated at 120 ^{o}C for 24 h. After cooling to room temperature, the powder is collected by centrifugation, washed with MeOH for three times, and then dried under vacuum at room temperature.

Fabrication of MOF-based MMMs with pre-synthesis method: Taking HKUST-1@PVDF-40 fabricated with pre-synthesis method for example: polymer powder (1.4 g) is completely dissolved in DMF solution (10 mL) and HKUST-1 powder (0.93 g) is added to the polymer solution under stirring at room temperature until it is well mixed. Then, a certain amount of solution is casted onto glass substrate with defined thickness using a doctor blade. After that, the glass substrate is transferred to an oven and heated at 120 ^{o}C for 30 min. After cooling to room temperature, the membrane is peeled off from the substrate followed with soaking in ethanol for several times and drying at 60 ^{o}C under vacuum to obtain HKUST-1@PVC-40 membrane.

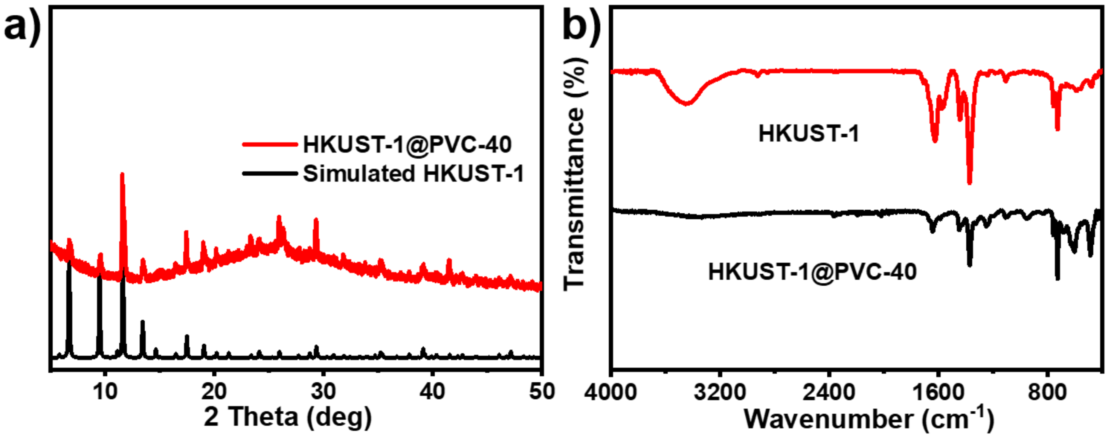

Figure S1. PXRD patterns and FT-IR spectra of HKUST-1@PVC-40 membrane from *in-situ* HASE method and reaction materials. a) PXRD patterns. b) FT-IR spectra.

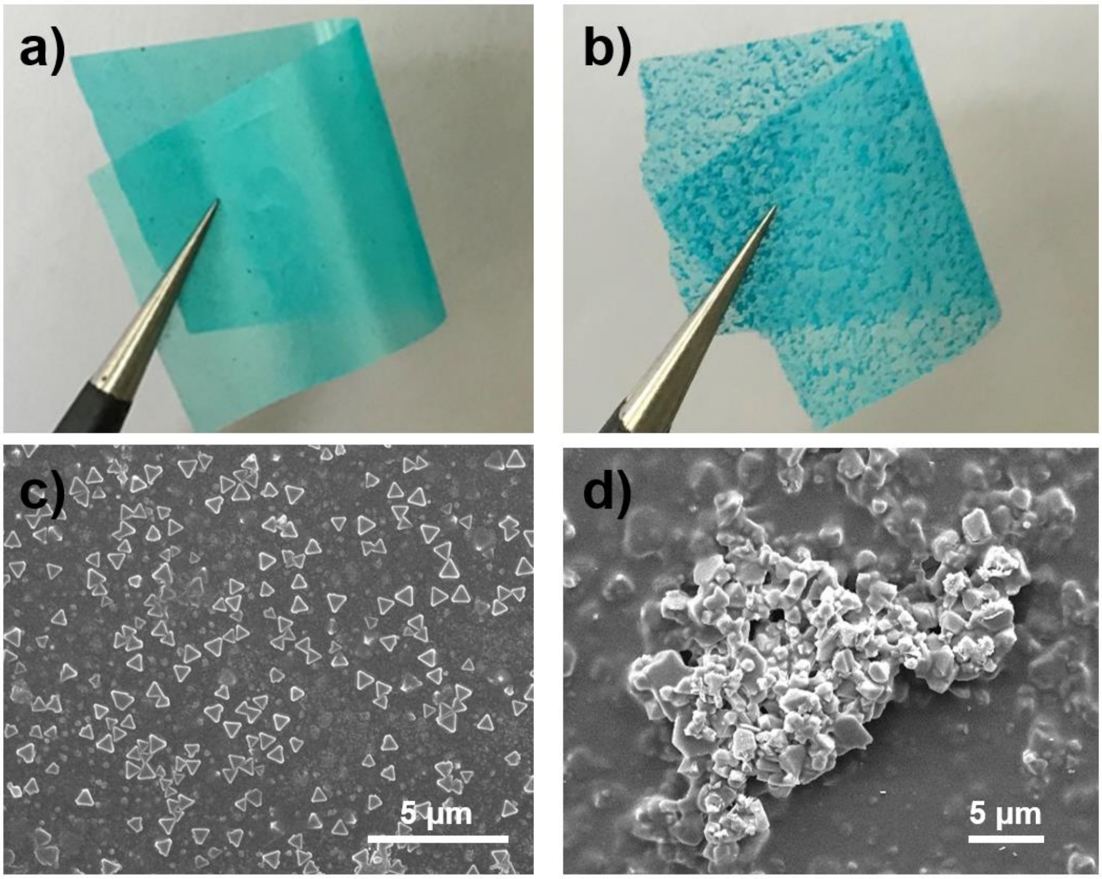

Figure S2. The membrane pictures and SEM images of HKUST-1@PVC-40 through *in-situ* HASE and pre-synthesis methods. a) HKUST-1@PVC-40 from *in-situ* HASE method. b) HKUST-1@PVC-40 from pre-synthesis method. c) SEM image of HKUST-1@PVC-40 from *in-situ* HASE method. d) SEM image of HKUST-1@PVC-40 membrane from pre-synthesis method. In *in-situ* HASE method, metal ions or organic ligands are uniformly mixed with polymers and polymers might serve as surfactant in controlling the growth of MOF particles and part of the polymer would also be inserted into the pore channel of MOFs, resulting in uniformly dispersed morphology and high robustness. For the physical mixing method, it would be difficult for the polymer segment to enter the MOF channel and provide sufficient interactions with MOFs, thus resulting in poorer dispersion and compatibility.

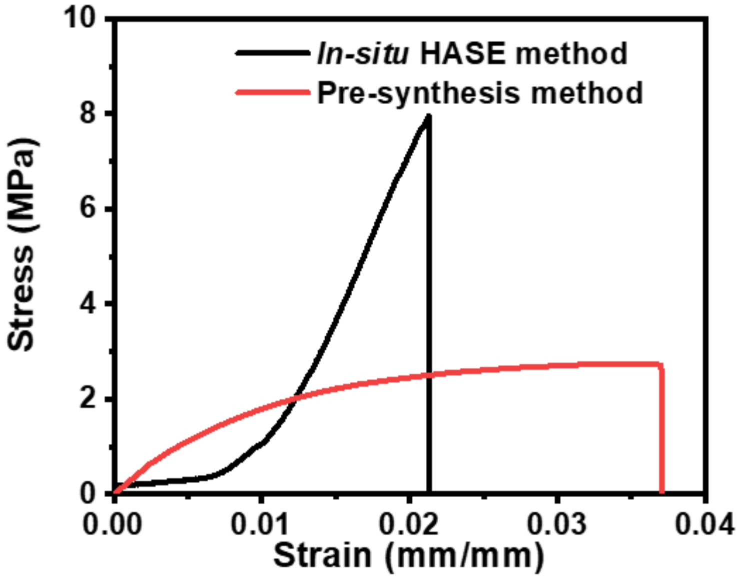

Figure S3. Stress-strain curves of HKUST-1@PVC-40 membranes fabrication through *in-situ* HASE and pre-synthesis methods. HKUST-1@PVC-40 (σ = ~7.93 MPa, ε = ~0.021%) shows higher tensile stress than pre-synthesized HKUST-1 based membrane with similar thickness (σ = ~2.74 MPa, ε = ~0.037%).

Table S1. A summary of the casting thickness, obtained thickness and HKUST-1 particle size of HKUST-1@PVC-40.

Casting thickness (μm)	Obtained thickness (μm)	Particle size (μm)
200	~40	0.13
400	~60	0.85
750	~80	1.76
1000	~80	2.32

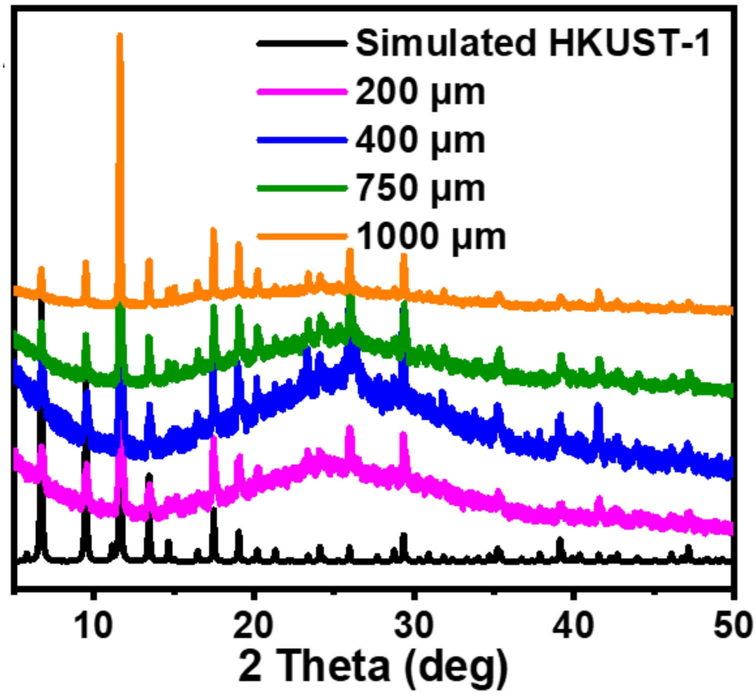

Figure S4. PXRD patterns of HKUST-1@PVC-40 with different thicknesses from *in-situ* HASE method.

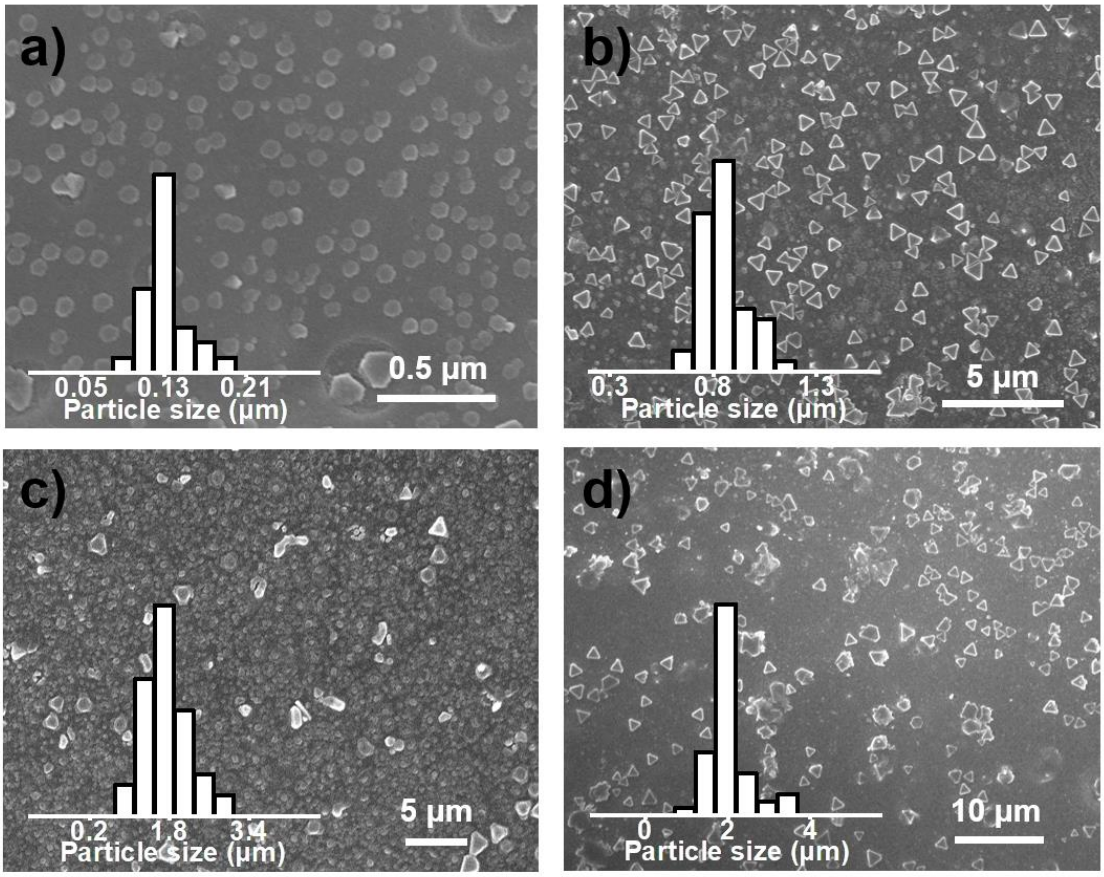

Figure S5. SEM images of HKUST-1@PVC-40 with different thicknesses and particle sizes (measured by Nano-measurer software) from *in-situ* HASE method. The membrane thickness is tuned using a doctor blade with pre-set thickness. a) 200 μm. b) 400 μm. c) 750 μm. d) 1000 μm.

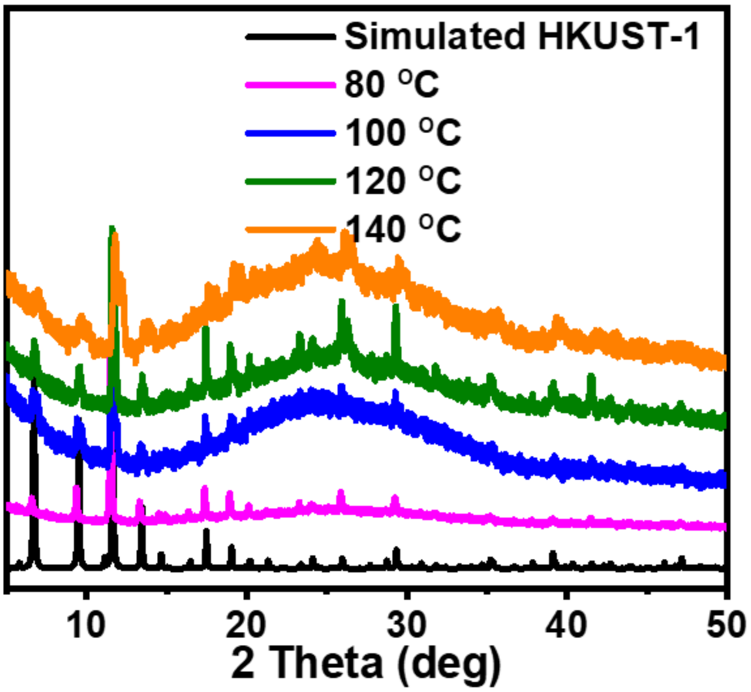

Figure S6. PXRD patterns of HKUST-1@PVC-40 with different fabrication temperatures from *in-situ* HASE method.

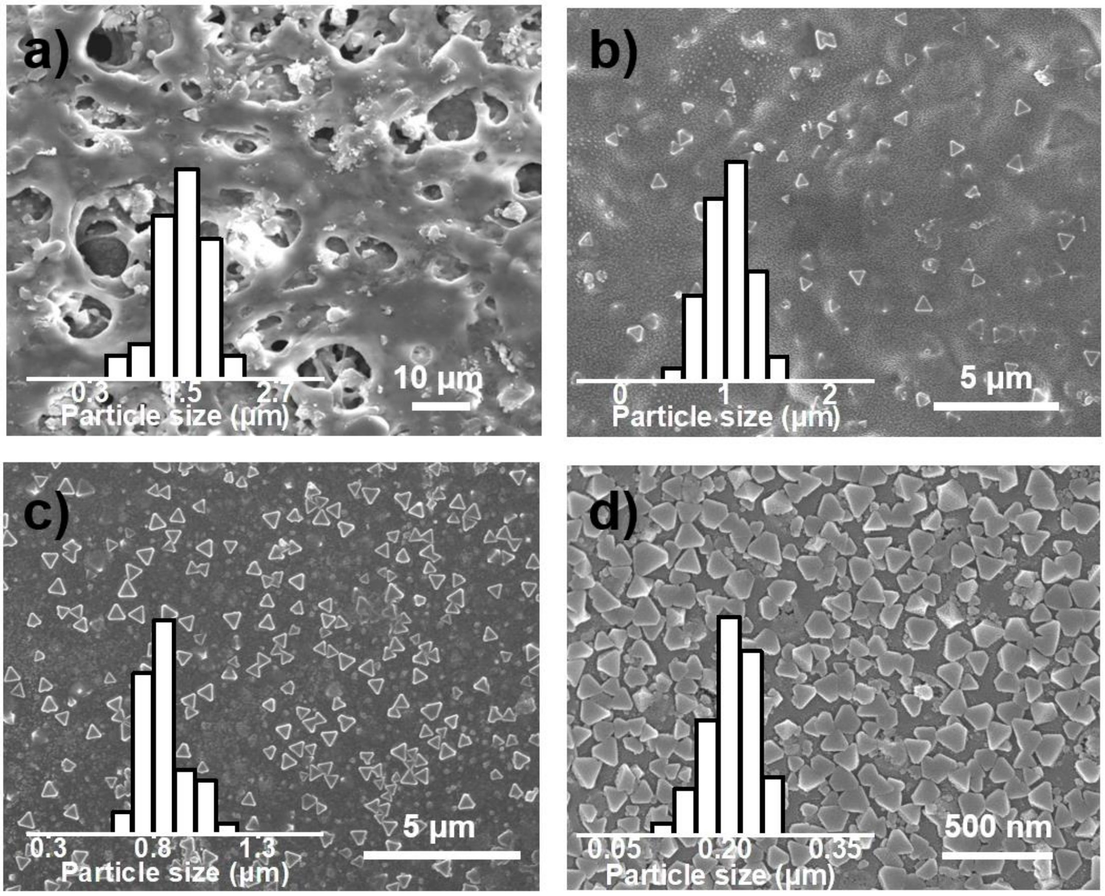

Figure S7. SEM images of HKUST-1@PVC-40 fabricated at diverse temperatures from *in-situ* HASE method. The particle size of HKUST-1@PVC related to temperature are: a) ~1.48 μm (80 ^{o}C). b) ~1.02 μm (100 ^{o}C). c) ~0.85 μm (120 ^{o}C). d) ~0.2 μm (140 ^{o}C).

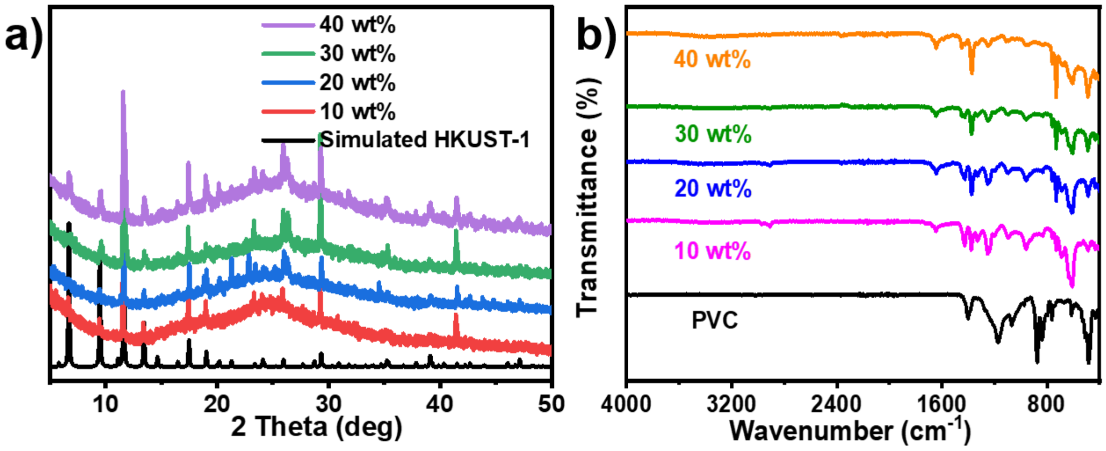

Figure S8. PXRD patterns and FT-IR spectra of HKUST-1@PVC with different loadings from *in-situ* HASE method. a) PXRD patterns. b) FT-IR spectra.

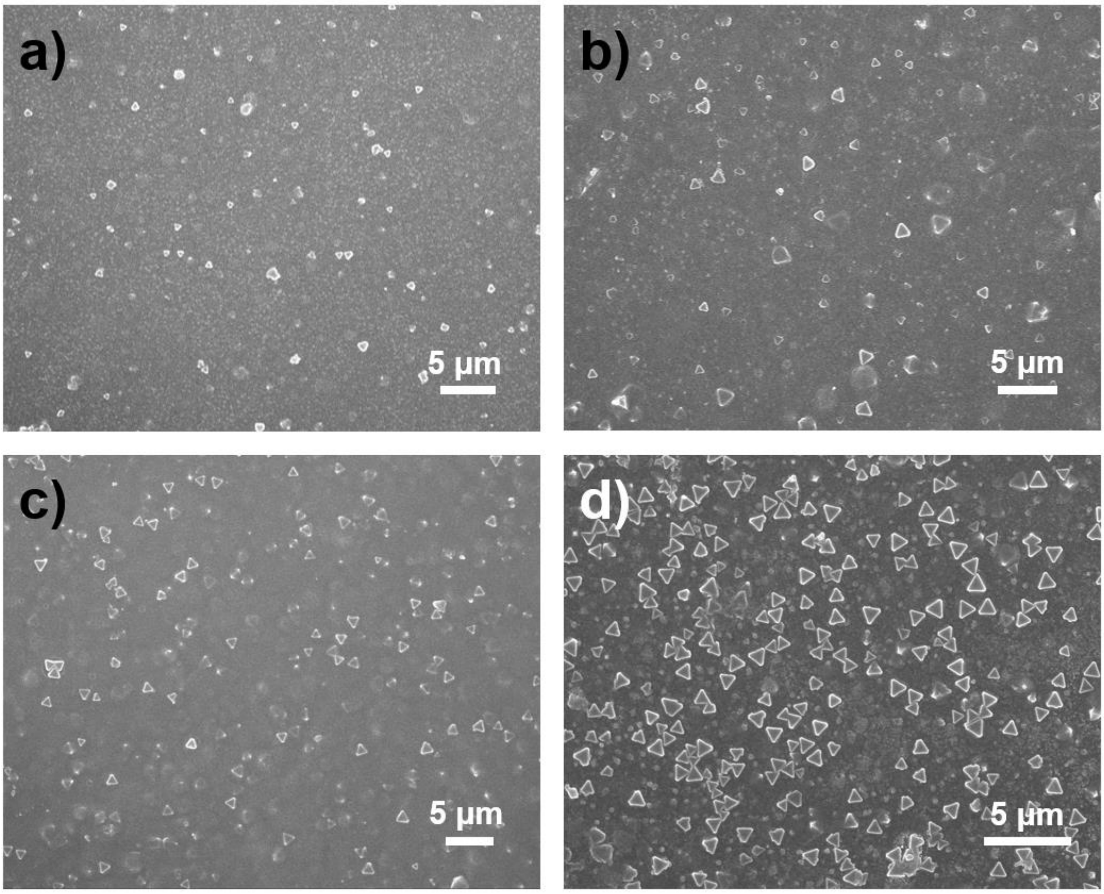

Figure S9. SEM images of HKUST-1@PVC with different MOF loadings from *in-situ* HASE method. a) 10 wt%. b) 20 wt%. c) 30 wt%. d) 40 wt%.

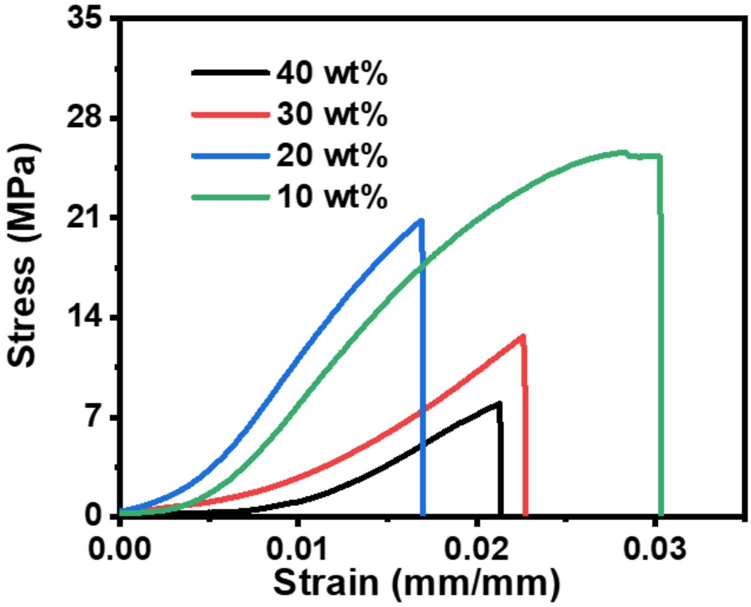

Figure S10. Stress-strain curves of HKUST-1@PVC with different loadings from *in-situ* HASE method.

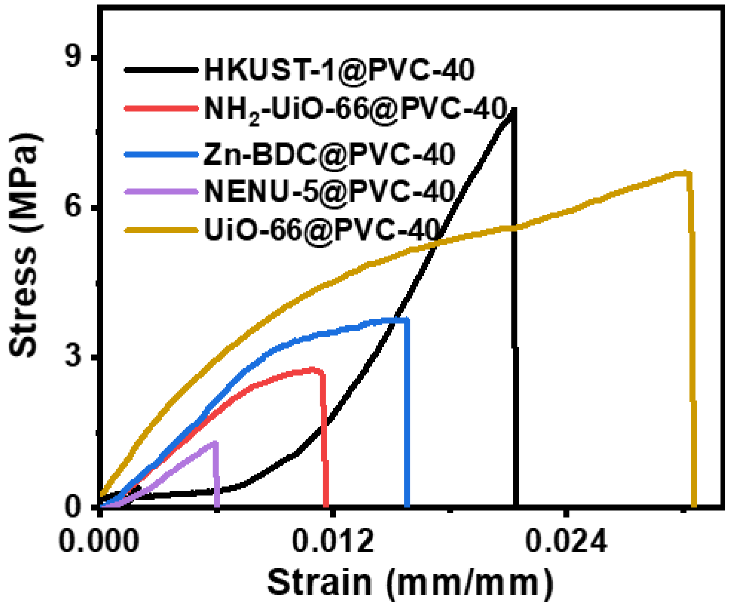

Figure S11. Stress-strain curves of various MOF@PVC-40 membranes from *in-situ* HASE method.

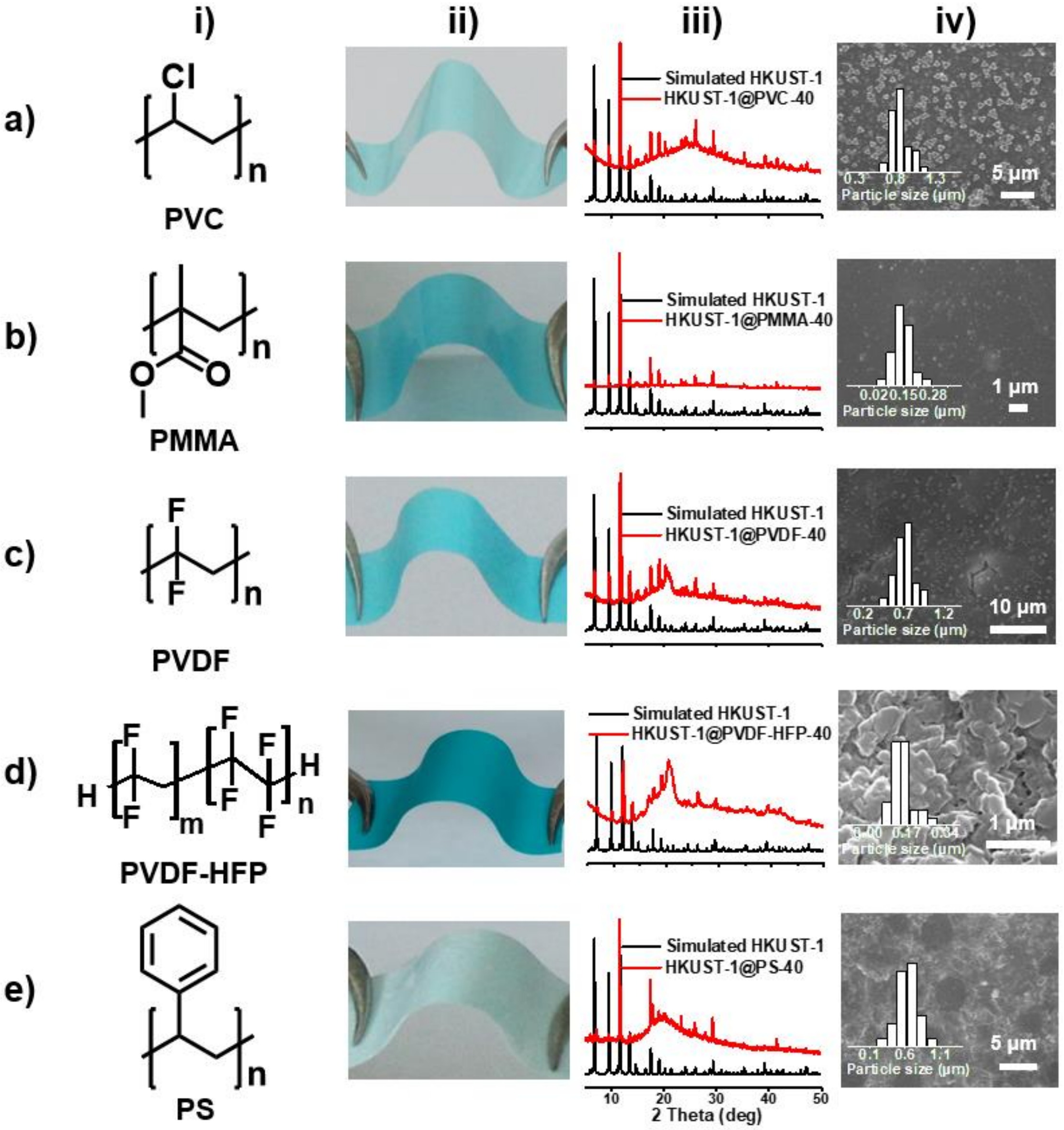

Figure S12. Structure, SEM images and PXRD patterns of the MOF-based MMMs (40 wt% loading) obtained from *in-situ* HASE method. a) HKUST-1@PVC-40. b) HKUST-1@PMMA-40. c) HKUST-1@PVDF-40. d) HKUST-1@PVDF-HFP-40. e) HKUST-1@PS-40. i) Structure images of MOFs. ii) Photo images of MOF-based MMMs. iii) PXRD patterns of MOF-based MMMs with different structures. iv) SEM images of the prepared MOF-based MMMs.

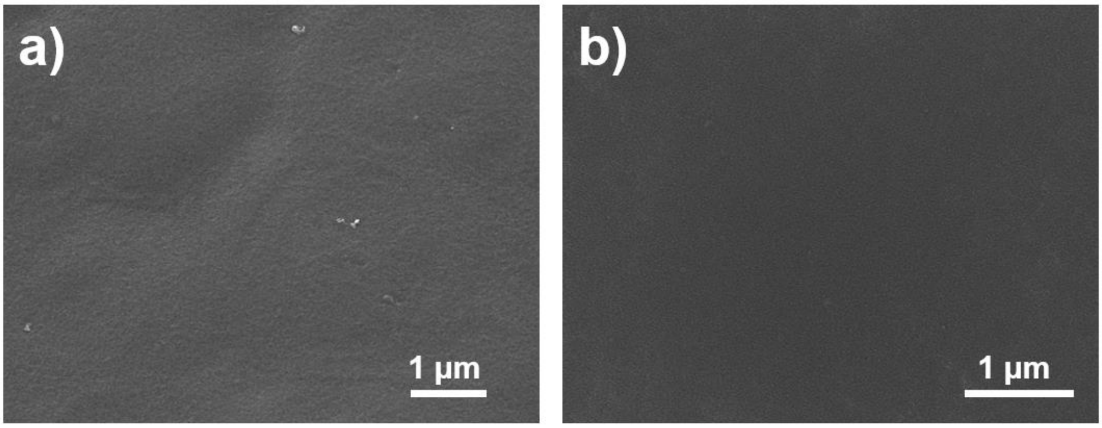

Figure S13. SEM of the top-view images with different polymer-based membranes from *in-situ* HASE method (doctor blade thickness is 400 μm). a) PVDF membrane. b) PVC membrane.

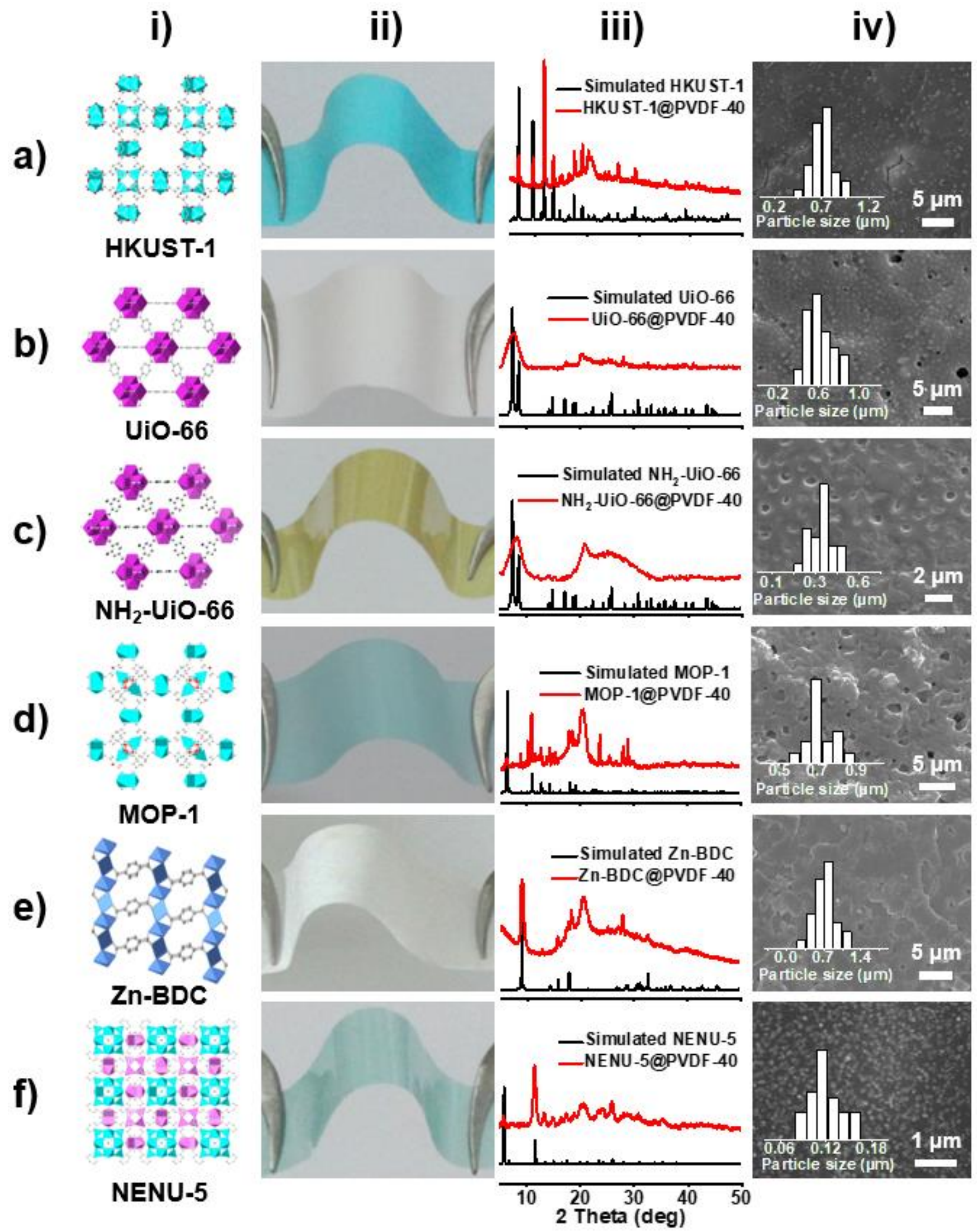

Figure S14. Structure, SEM images and PXRD patterns of the MOF-based membranes (40 wt% loading) obtained from *in-situ* HASE method. a) HKUST-1@PVDF-40. b) UiO-66@PVDF-40. c) NH_2-UiO-66@PVDF-40. d) MOP-1@PVDF-40. e) Zn-BDC@PVDF-40. f) NENU-5@PVDF-40. i) Structures images of MOFs. ii) Photo images of MOF-based MMMs. iii) PXRD patterns of MOF-based MMMs with different structures. iv) SEM images of the prepared MOF-based MMMs.

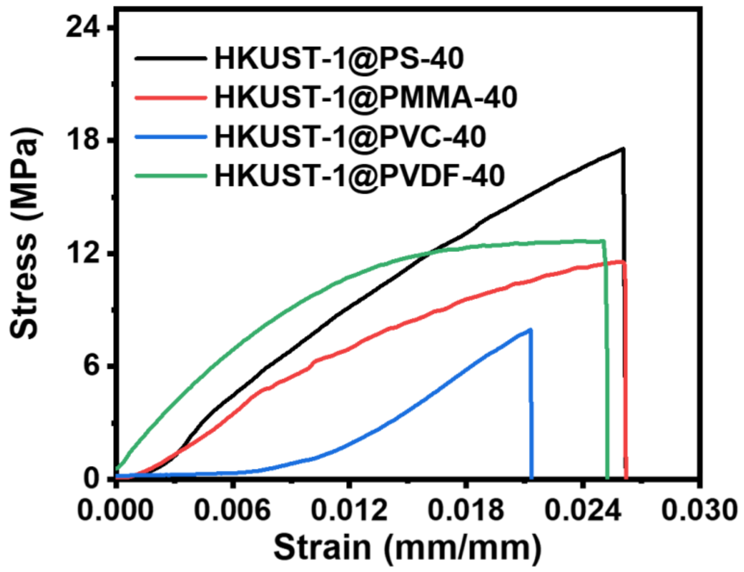

Figure S15. Stress-strain curves of various HKUST-1@Polymer-40 from *in-situ* HASE method.

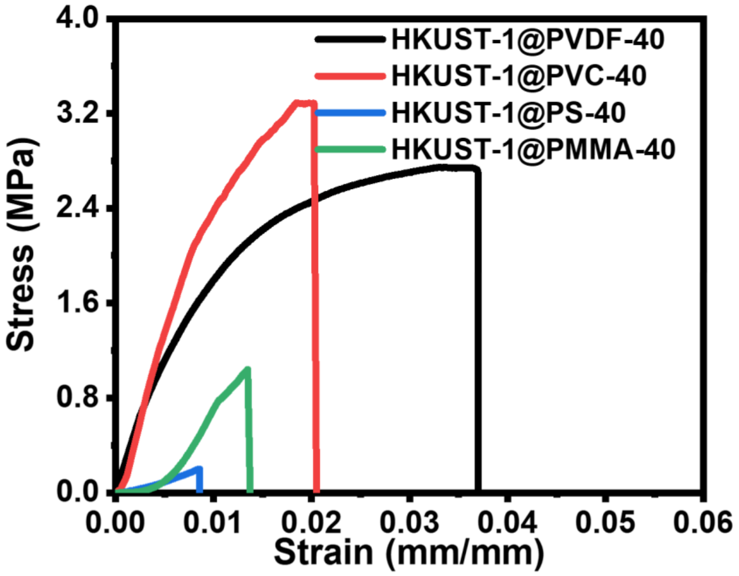

Figure S16. Stress-strain curves of HKUST-1 based different polymer membranes fabricated from pre-synthesis method.

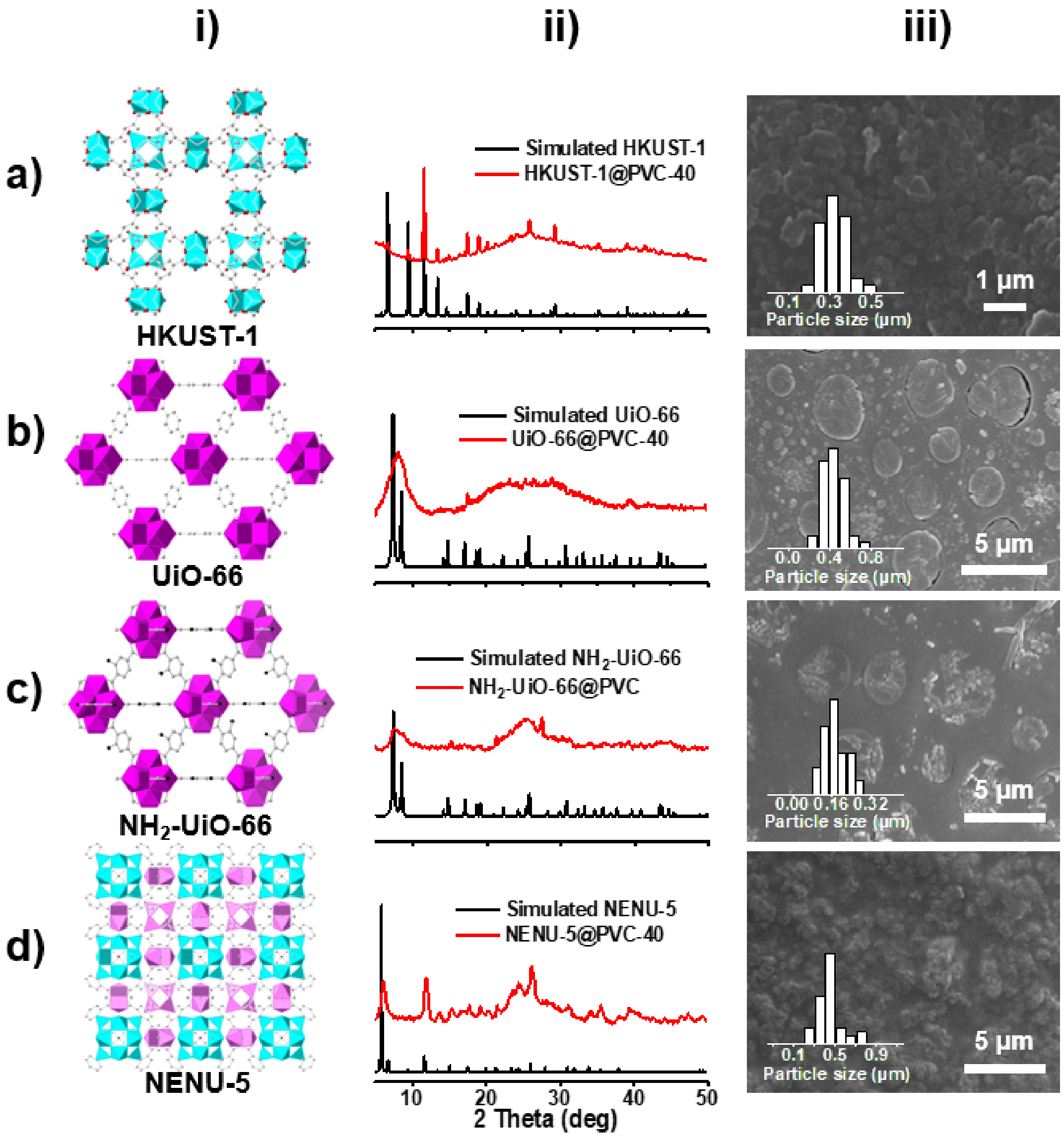

Figure S17. Structure, SEM images and PXRD patterns of the MOF-based MMMs obtained from microwave-assisted method (40 wt% loading). a) HKUST-1@PVC-40. b) UiO-66@PVC-40. c) NH_2-UiO-66@PVC-40. d) NENU-5@PVC-40. i) Structure images of MOFs. ii) PXRD patterns of MOF-based MMMs with different structures. iii) SEM images of MOF-based MMMs.

Table S2. A summary of the average particle size of HKUST-1 nanoparticle through oven-heating and microwave-assisted methods.

Polymer	Membrane	Oven-heating (μm)	Microwave-assisted (μm)
PVC	HKUST-1@PVC-40	0.85	0.27
	UiO-66@PVC-40	0.97	0.42
	NH_2-UiO-66@PVC-40	0.70	0.18
	NENU-5@PVC-40	0.82	0.46
PVDF	HKUST-1@PVDF-40	0.79	0.80
	UiO-66@PVDF-40	0.62	0.43

NH_2-UiO-66@PVDF-40	0.39	0.40
NENU-5@PVDF-40	1.06	0.51

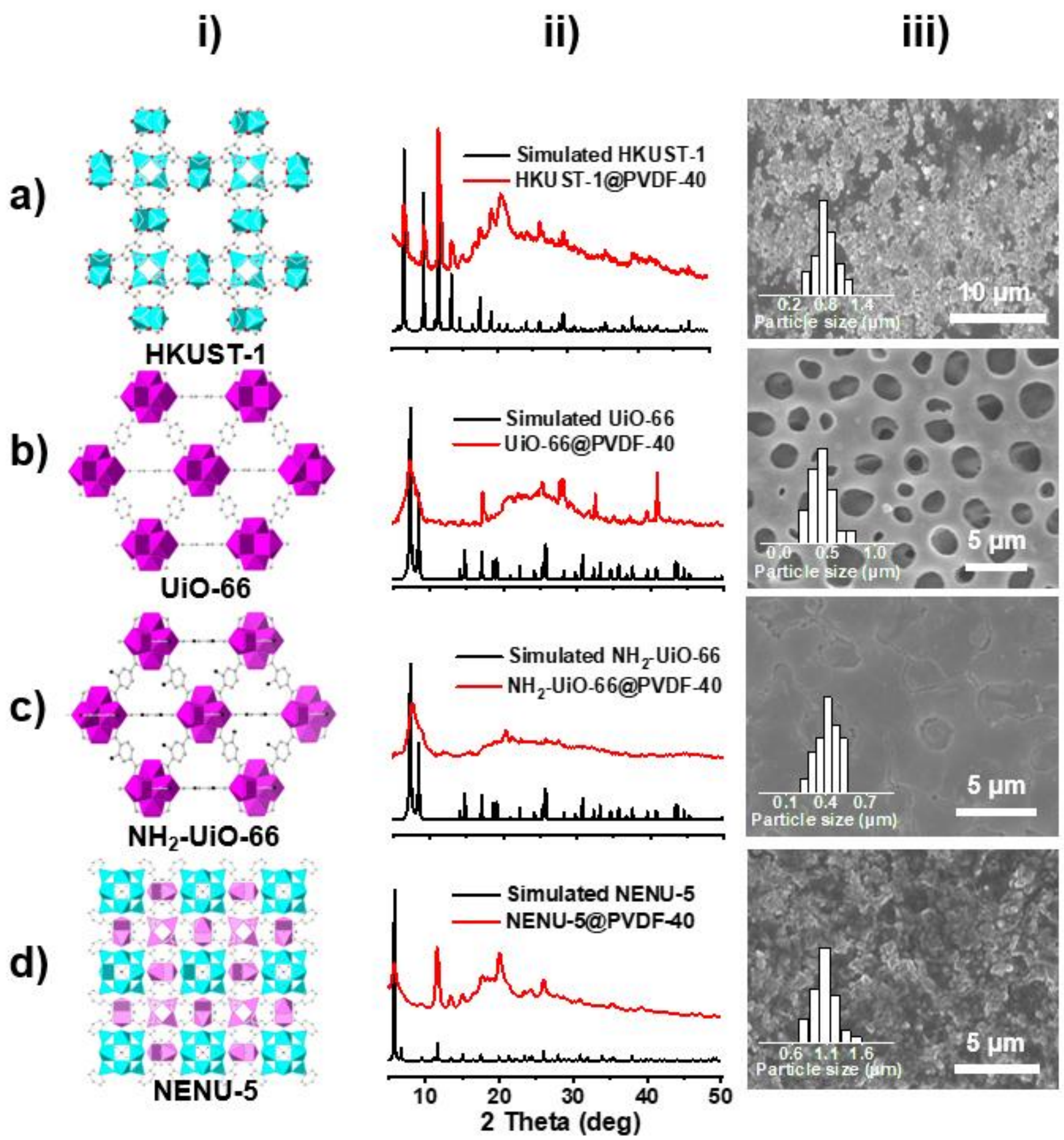

Figure S18. Structure, SEM images and PXRD patterns of the MOF-based MMMs (40 wt% loading) obtained from microwave-assisted method. a) HKUST-1@PVDF-40. b) UiO-66@PVDF-40. c) NH_2-UiO-66@PVDF-40. d) NENU-5@PVDF-40. i) Structure images of MOFs. ii) PXRD patterns of MOF-based MMMs with different structures. iii) SEM images of the prepared MOF-based MMMs.

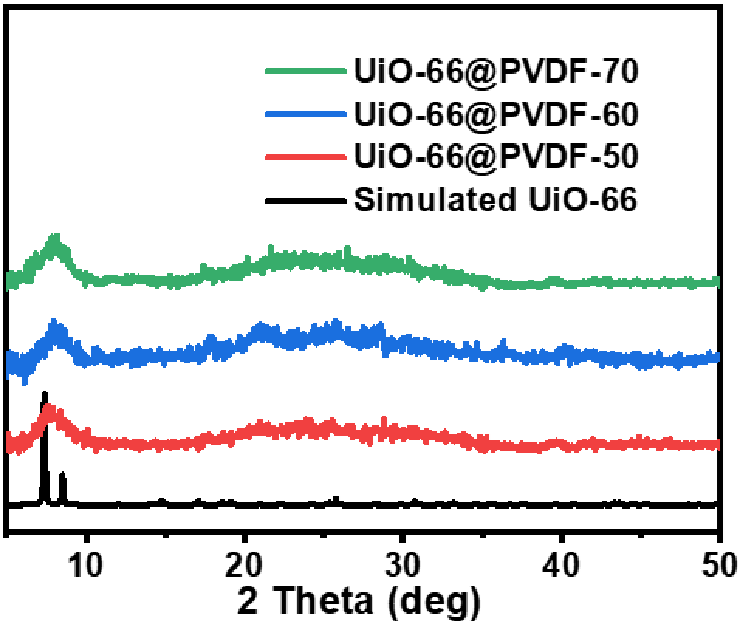

Figure S19. PXRD patterns of UiO-66@PVDF with different loadings from *in-situ* HASE method.

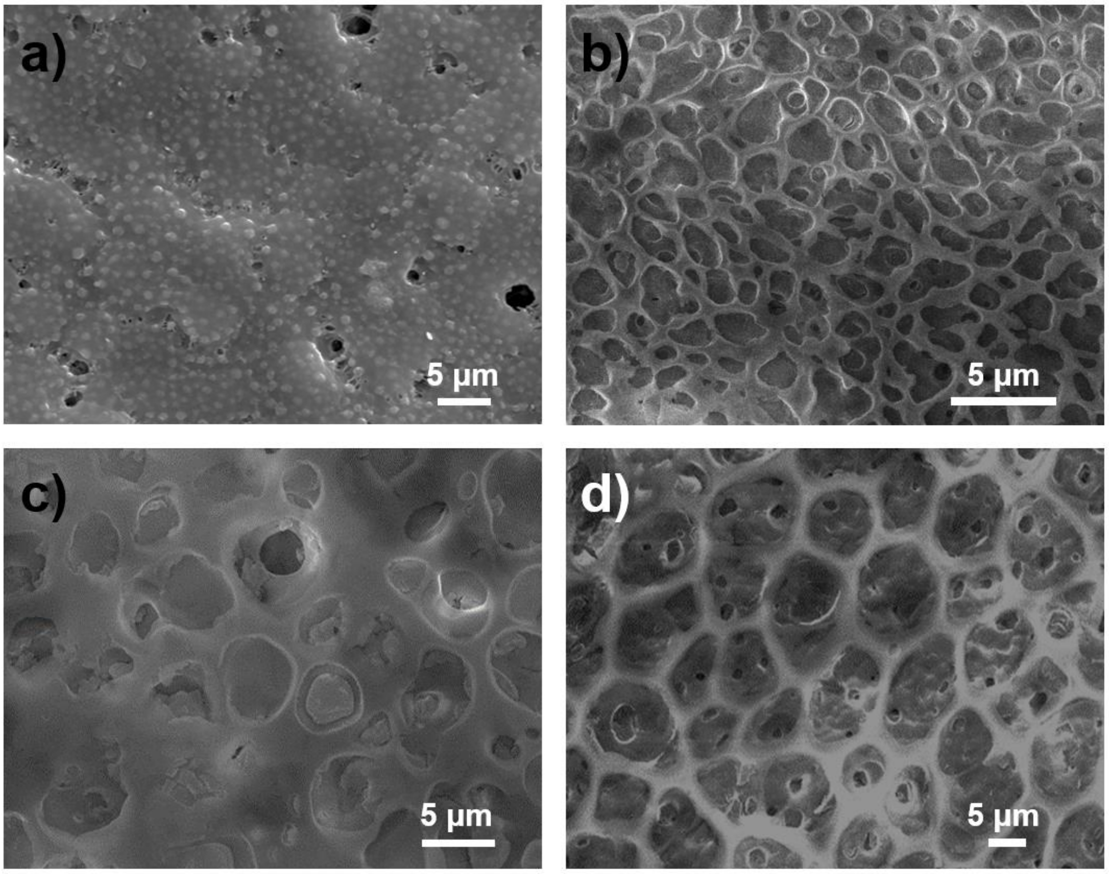

Figure S20. SEM images of the top-view for UiO-66@PVDF with different loadings from *in-situ* HASE method. a) UiO-66@PVDF-40. b) UiO-66@PVDF-50. c) UiO-66@PVDF-60. d) UiO-66@PVDF-70.

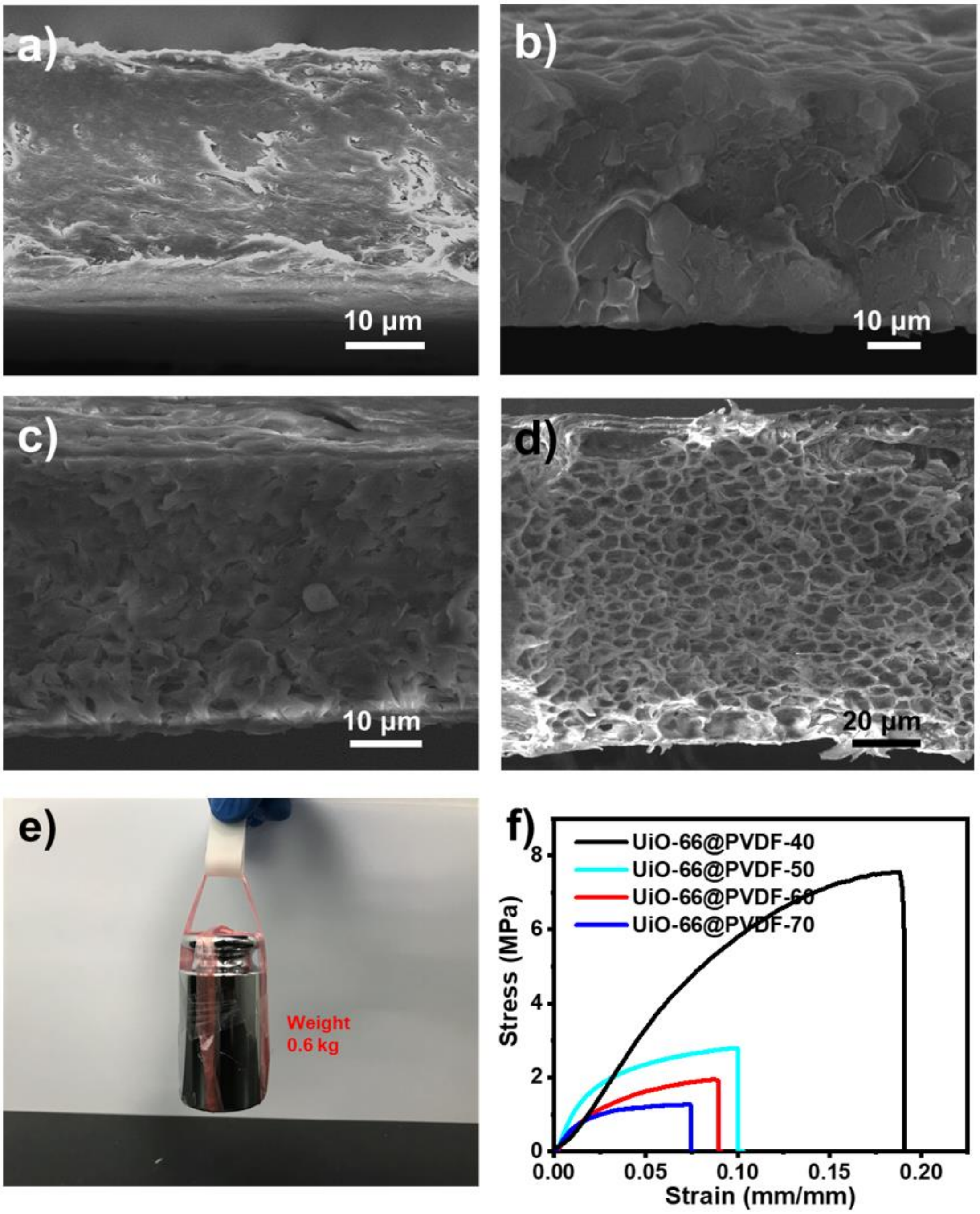

Figure S21. SEM images of the cross section for the different loading membranes and tensile test of UiO-66@PVDF-70 from *in-situ* HASE method. a) UiO-66@PVDF-40. b) UiO-66@PVDF-50. c) UiO-66@PVDF-60. d) UiO-66@PVDF-70. e) Tensile experiment of the UiO-66@PVDF-70 (width, ~1.5 cm) conducted with a weight. f) Stress-strain curve of UiO-66@PVDF with different loadings.

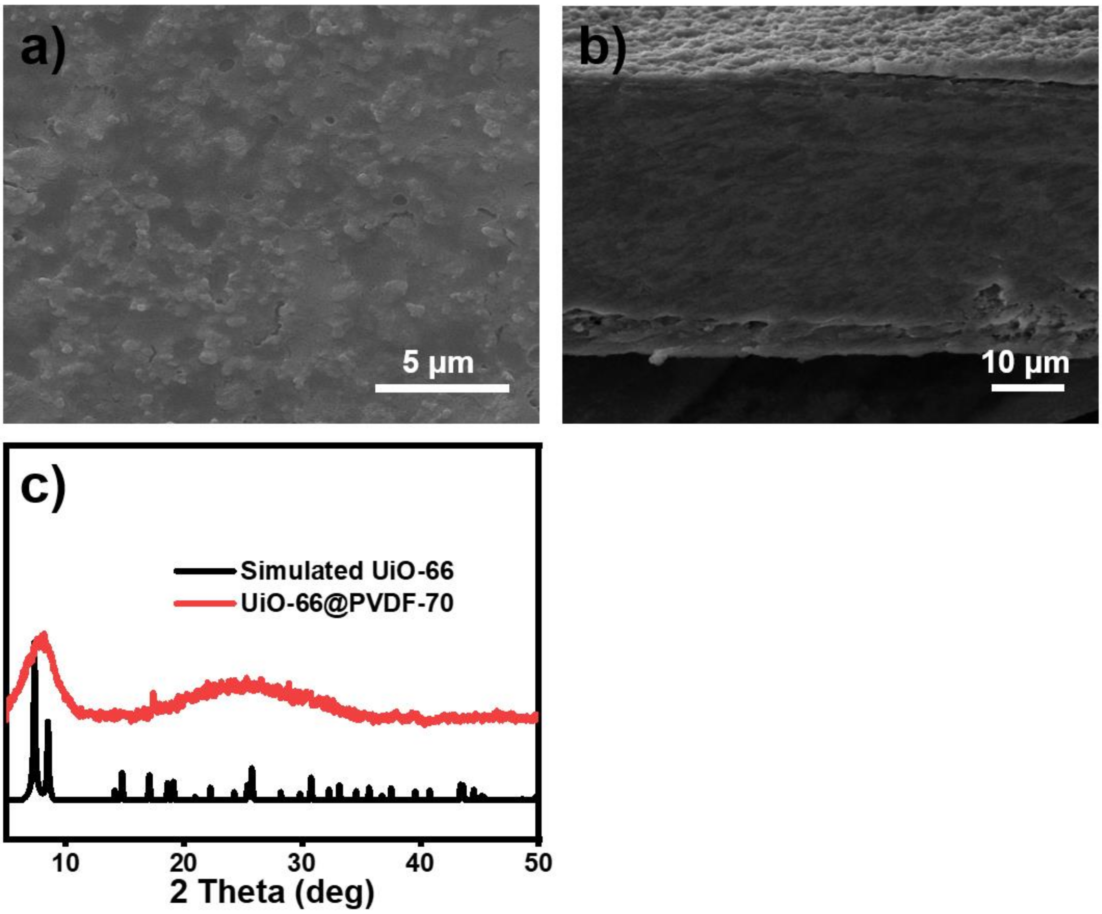

Figure S22. SEM images and PXRD patterns of UiO-66@PVDF-70 through microwave-assisted method. a) The SEM image for the top-view of UiO-66@PVDF-70. b) The SEM image for the cross section of UiO-66@PVDF-70. c) The PXRD pattern for UiO-66@PVDF-70.

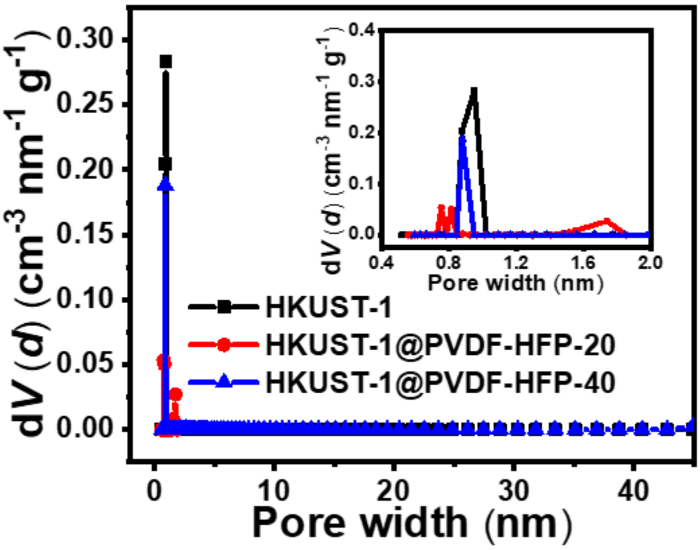

Figure S23. Pore size distribution of HKUST-1, HKUST-1@PVDF-HFP-20 and HKUST-1@PVDF-HFP-40. Insert image is the enlarged place from 0.4 to 2.0 nm.

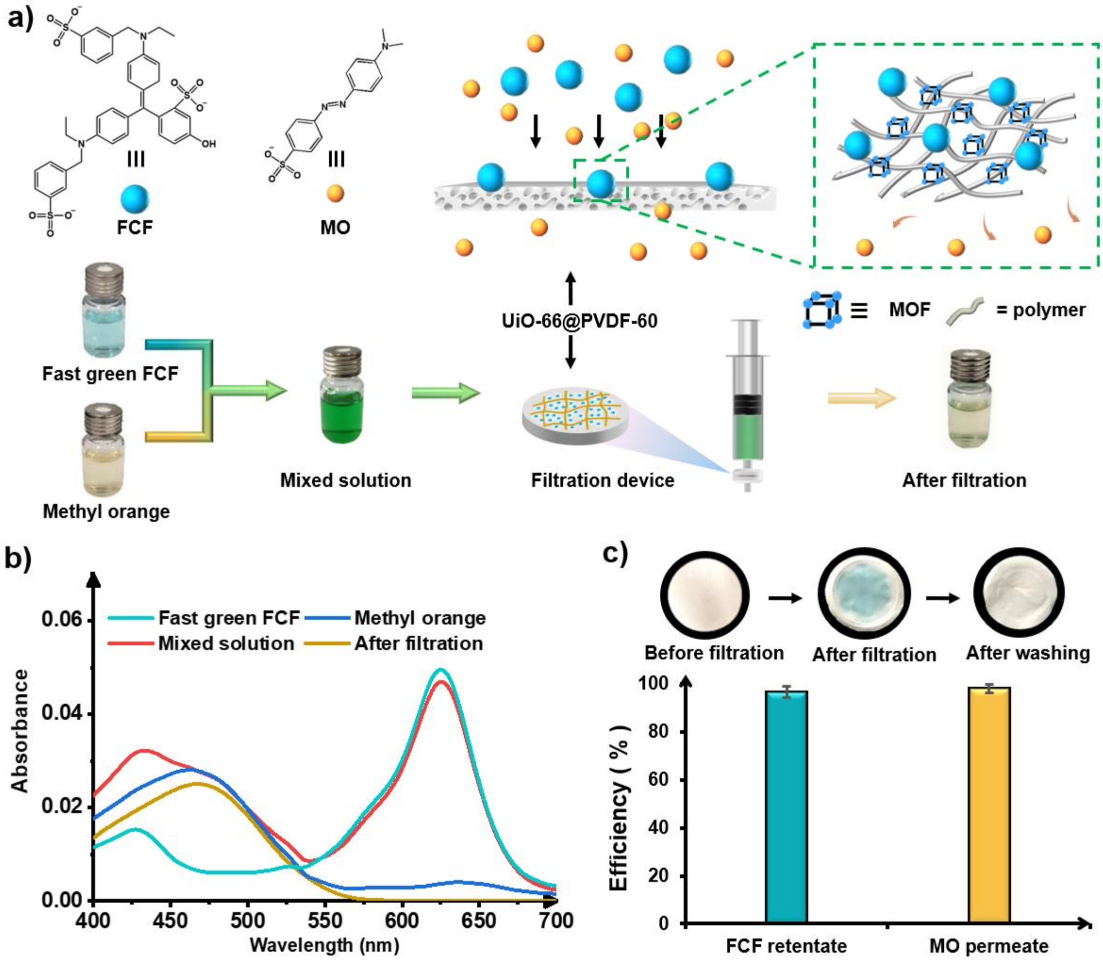

Figure S24. The filtration performance of UiO-66@PVDF-60. a) Molecular structure and size of FCF (17.8 Å × 14.5 Å) and MO (14.9 Å × 4.3 Å). Schematic diagram of filtration device. The mixture of FCF (blue solution, 12 μM) and methyl orange (MO) (light yellow solution, 30 μM) results in a green solution, after filtration through UiO-66@PVDF-60, the color of the solution changes to light yellow similar as MO solution. b) UV-Vis spectra of FCF, MO, mixed solution and the mixed after solution filtration. c) Pictures of UiO-66@PVDF-60 before and after filtration, and after washing. The retentate efficiency of FCF and permeate efficiency of MO after filtration. The thermal stability of the MOF-based MMMs are determined by MOFs and polymers. The thermal stability of MOFs are mostly > 300 ^{o}C (e.g., HKUST-1, 350 ^{o}C[2]; UiO-66, 300 ^{o}C[3] and NH_2-UiO-66, 315 ^{o}C[4]). However, the melting points for most of polymers applied in this work are < 200 ^{o}C (melting point: PVDF, ~172 ^{o}C; PVC, ~85 ^{o}C and PS, ~166 ^{o}C). Therefore, the thermal stability of the MOF-based MMMs depends on the polymers and the applicable temperatures should take the thermal stability of polymers into consideration.

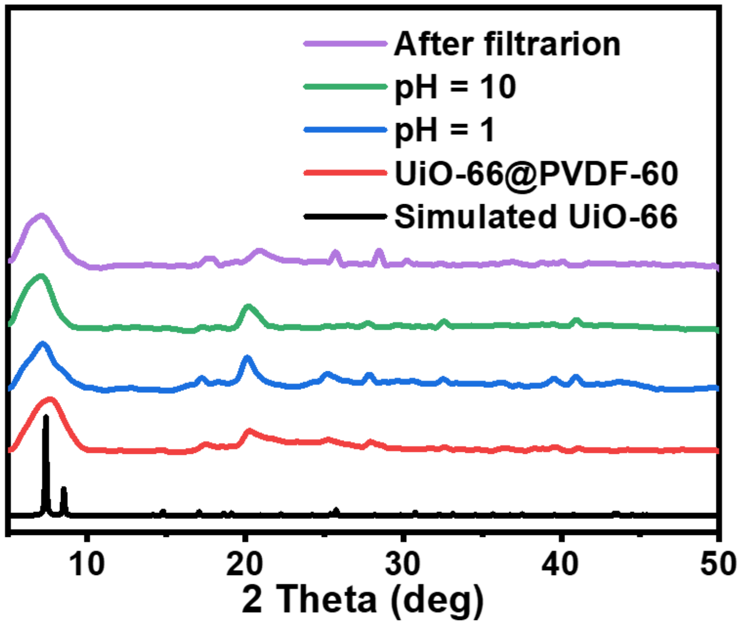

Supplementary Figure 25. PXRD patterns of the chemical and recycle stability for UiO-66@PVDF-60.

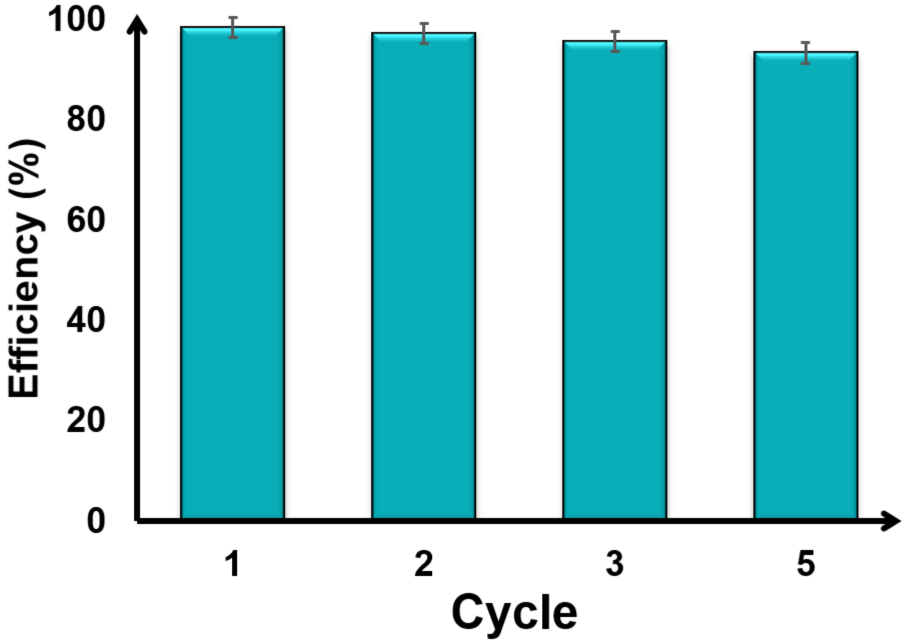

Figure S26. Recycle performances of UiO-66@PVDF-60 in the filtration of FCF.

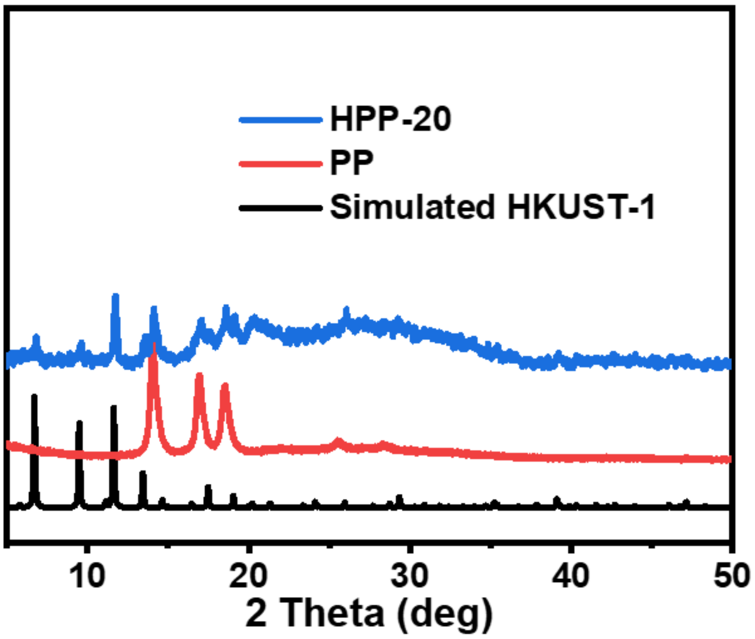

Figure S27. PXRD patterns of the HPP-20 separator.

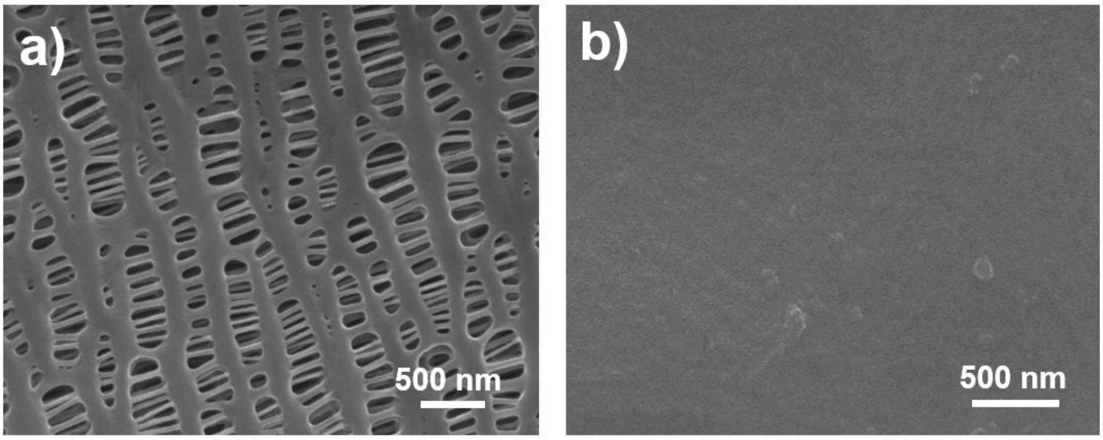

Figure S28. SEM of the top-view for different separators. a) The top-view of PP. b) The top-view of HPP-20.

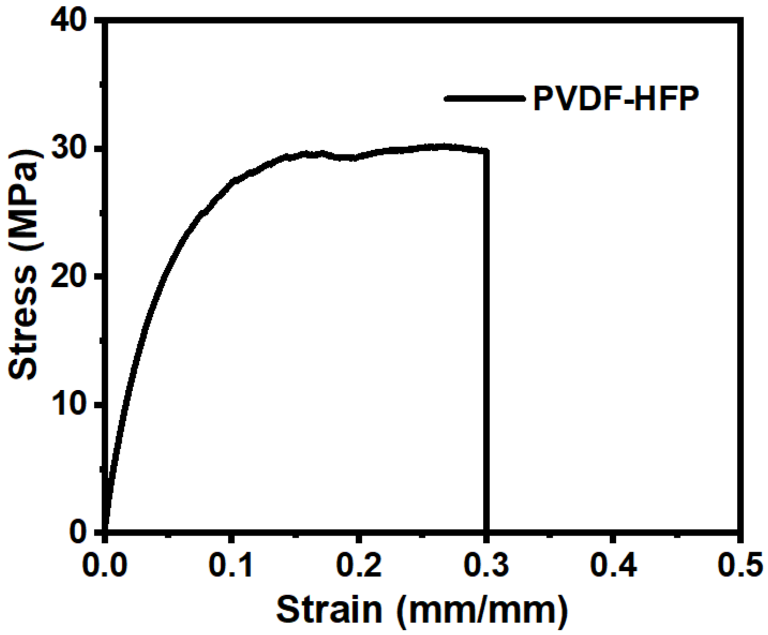

Supplementary Figure 29. Stress-strain curve of PVDF-HFP based membrane.

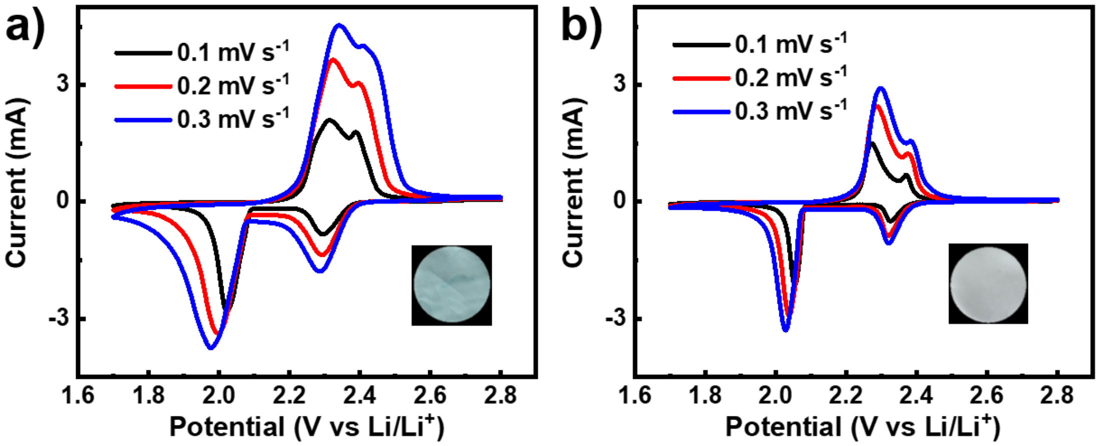

Figure S30. Photo images and cyclic voltammograms of different separators at various scan rates for Li-S cells. a) HPP-20. b) PP.

Table S3. A summary of the performances for Li-S cell separator obtained through different methods.

	Separator	MOF type	Additive	Method	S content (mg cm^{-2})	Capacity	Ref
1	Celgard 2400	$Ni_3(HITP)_2$	Ammonium hydroxide	Interface-induced growth	3.5	1244 to1139 mA h g^{-1}, 0.2 C, 100 cycles	5
2	Celgard 2400	ZIF-8	Ammonium hydroxide	Interface-induced growth	3.5	945 mA h g^{-1}, 0.2 C, 100 cycles	5
3	Celgard 2500	UiO-66	PVDF, Super P	Casting	1.5	1032 to 720 mA h g^{-1}, 0.5 C, 500 cycles	6

4	None	HKUST-1	GO	Vacuum filtration	0.6~0.8	1126 to 799 mA h g^{-1}, 0.5 C ,500 cycles	7
5	None	Zn-HKUST-1	GO	Vacuum filtration	0.6~0.8	685mA h g^{-1}, 1 C, 350 cycles, 657 mA h g^{-1}, 1 C, 1000 cycles	8
6	None	HKUST-1	PVDF-HFP	Vacuum filtration	1~1.5	1196 to 802 mA h g^{-1}, 0.5 C, 600 cycles	9
7	Celgard 2400	HKUST-1	PSS	Vacuum filtration	1.3	1278 to 775 mA h g^{-1}, 0.5 C, 500 cycles	10
8	Glass fiber	HKUST-1	PVDF, Carbon nanotubes	Vacuum filtration	~1	1032 to 197 mA h g^{-1}, 0.25 C, after 300 cycles	11
9	Glass fiber	ZIF-8	PVDF, Carbon nanotubes	Vacuum filtration	~1	925 to 403 mA h g^{-1}, 0.25 C, after 300 cycles	11
10	Glass fiber	ZIF-7	PVDF, Carbon nanotubes	Vacuum filtration	~1	989 to 452 mA h g^{-1}, 0.25 C, after 300 cycles	11
11	Glass fiber	Y-FTZB	PVDF, Carbon nanotubes	Vacuum filtration	~1	1101 to 557 mA h g^{-1}, 0.25 C, after 300 cycles	11
12	PE	Ni-MOF	Multi-walled carbon nanotubes	Vacuum filtration	2	1183 mA h g^{-1}, 0.2 C, after 300 cycles	12
13	Celgard 2400	Ce-MOF	CNT, LA132, Super P	Coating	2.5	1021.8 to 838.8 mA h g^{-1}, 1 C, after 800 cycles	13
14	Celgard 2500	ZIF-8	MWCNTs, PVDF	Casting	~1.2	1588.4 to 870.3 mA h g^{-1}, 0.2 C, after 100 cycles	14
15	PVDF (GPE)	Mg-MOF-74	Polyvinylpyrrolidone	Vacuum filtration	0.8~1.2	1383.1 to 981.1 mA h g^{-1}, 1 C, 200 cycles	15
16	PP	$Ni_3(HITP)_2$	Ammonium hydroxide, PVDF	Vacuum filtration	-	585.4 mA h g^{-1}, 0.5 C, after 300 cycles	16
17	Celgard PP	$Cu_2(CuTCPP)$ nanosheets	LA133	Vacuum filtration	2	850 to 604 mA h g^{-1}, 0.05 C, after 900 cycles	17
18	PE	UiO-66-SO_3	Nafion	Vacuum filtration	1.7	1127.4 to 872.3 mA h g^{-1}, 0.1 C, 200 cycles	18
19	Al_2O_3/PP	Cu-BTC	Sulfonic polymer, PVDF	Uniformly spread	2.4	1279 mAh g^{-1} to 1000 mAh g^{-1}, 1 C, 1000 cycles	19
20	Celgard 2400	HKUST-1	PVDF-HFP	*In-situ* HASE	0.8-1.2	1163.7 to 500.7 mA h g^{-1}, 0.5 C, 700 cycles	This work

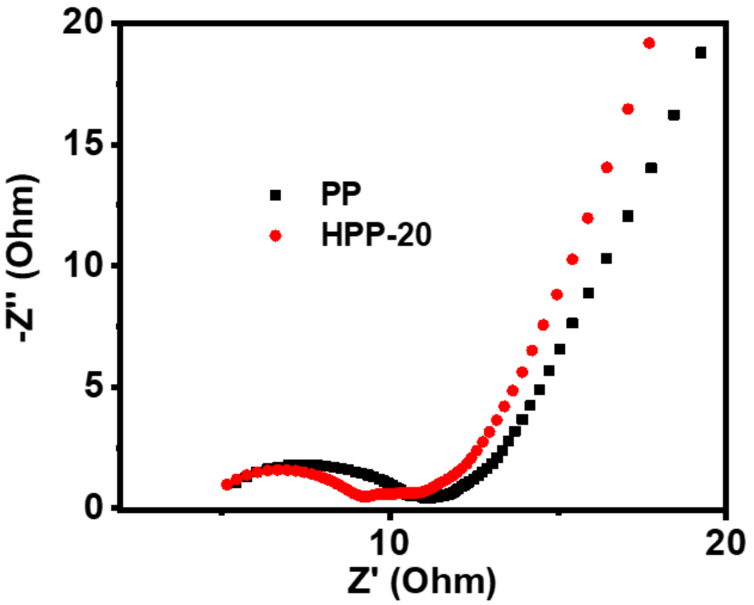

Figure S31. The electrochemical impedance spectra of Li-S coin cells with different separators. The second peak for HPP-20 might be attributed to the charge transfer resistance between PP and MMM, which is absent for PP.[20]

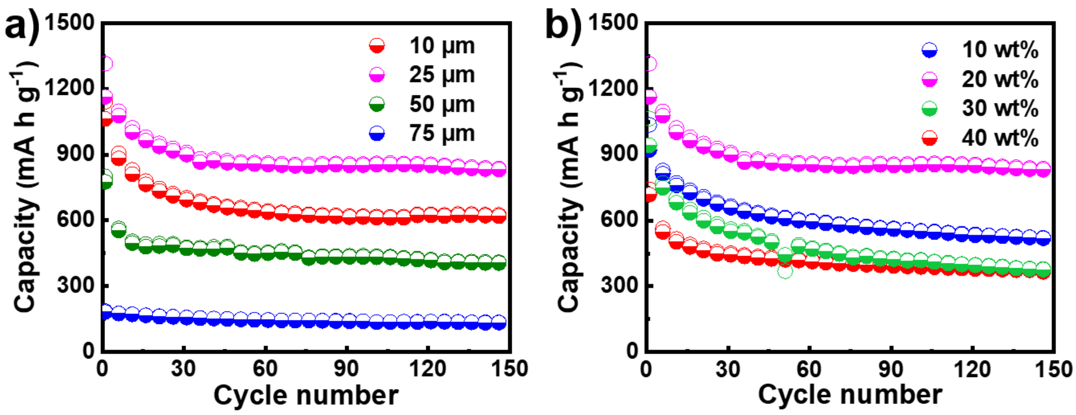

Figure S32. Long life cycle tests of HPP with different thicknesses and loadings in Li-S cells. a) Different thicknesses. b) Different loadings.

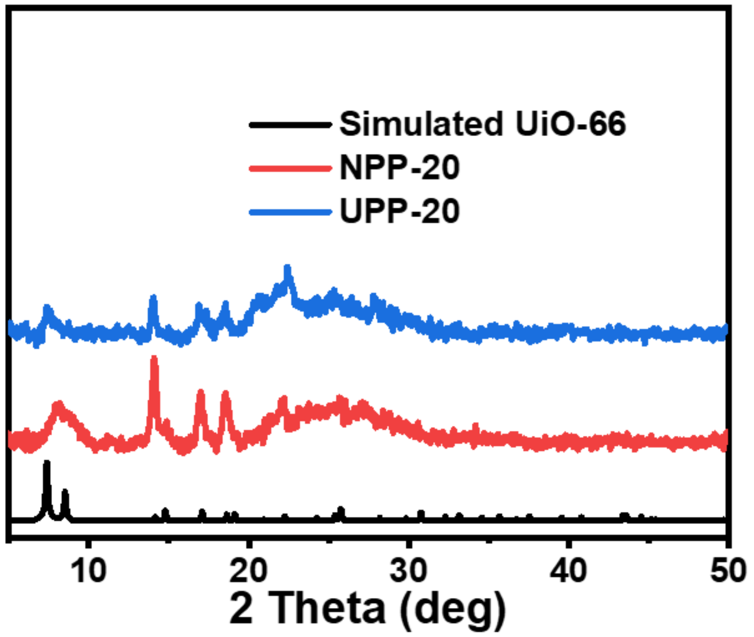

Figure S33. PXRD patterns of the UPP-20 and NPP-20.

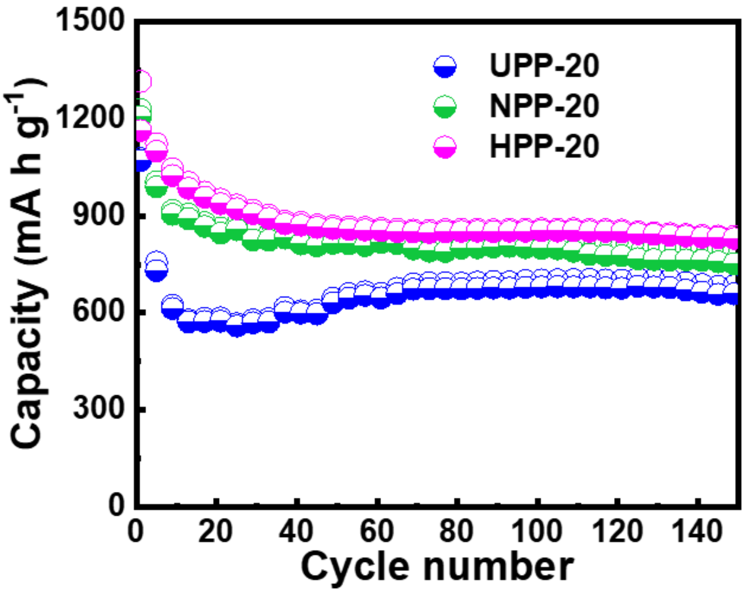

Figure S34. Long life cycle tests of UPP-20 and NPP-20 based Li-S cells.

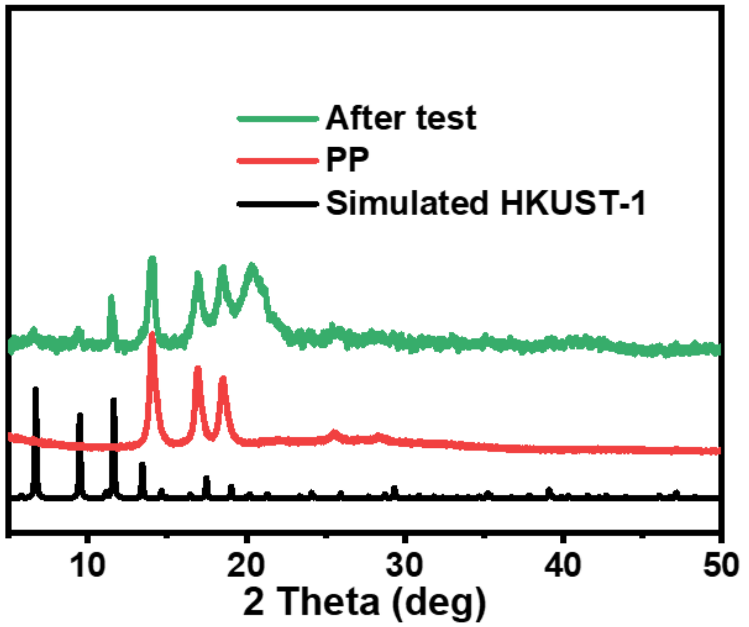

Figure S35. PXRD patterns of the HPP-20 after test.

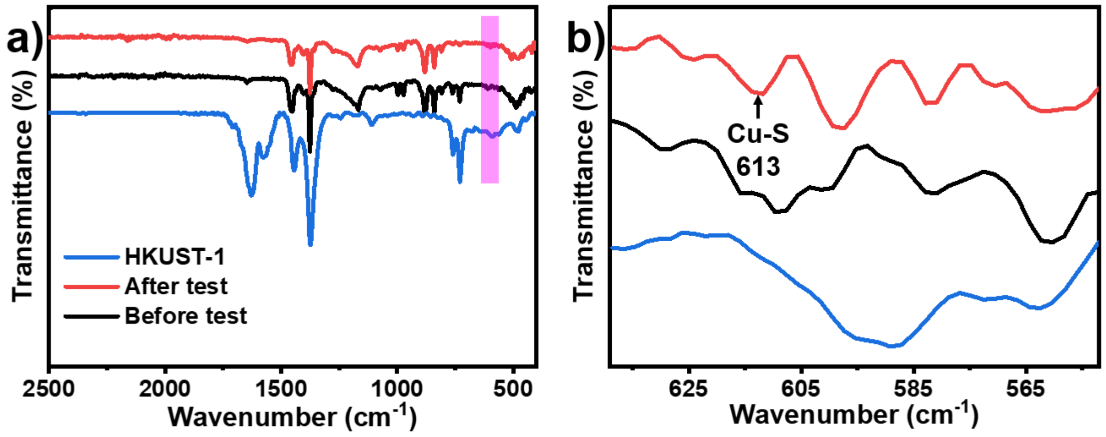

Figure S36. FT-IR spectra of HPP-20. a) Before and after test. b) is the enlarged pink place in a).

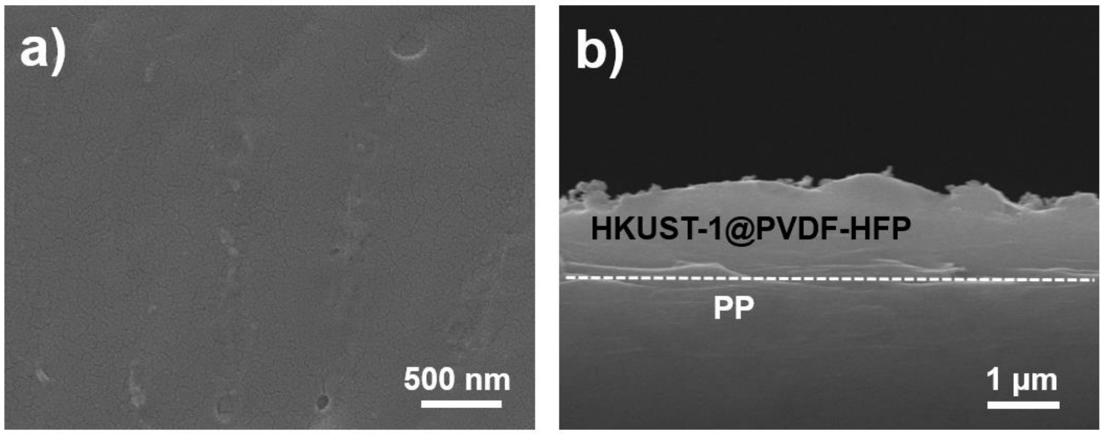

Figure S37. SEM of the top-view and cross section for HPP-20 after test. a) The top-view image. b) The cross section image. The thickness of HKUST-1@PVDF-HFP is ~1.2 μm.

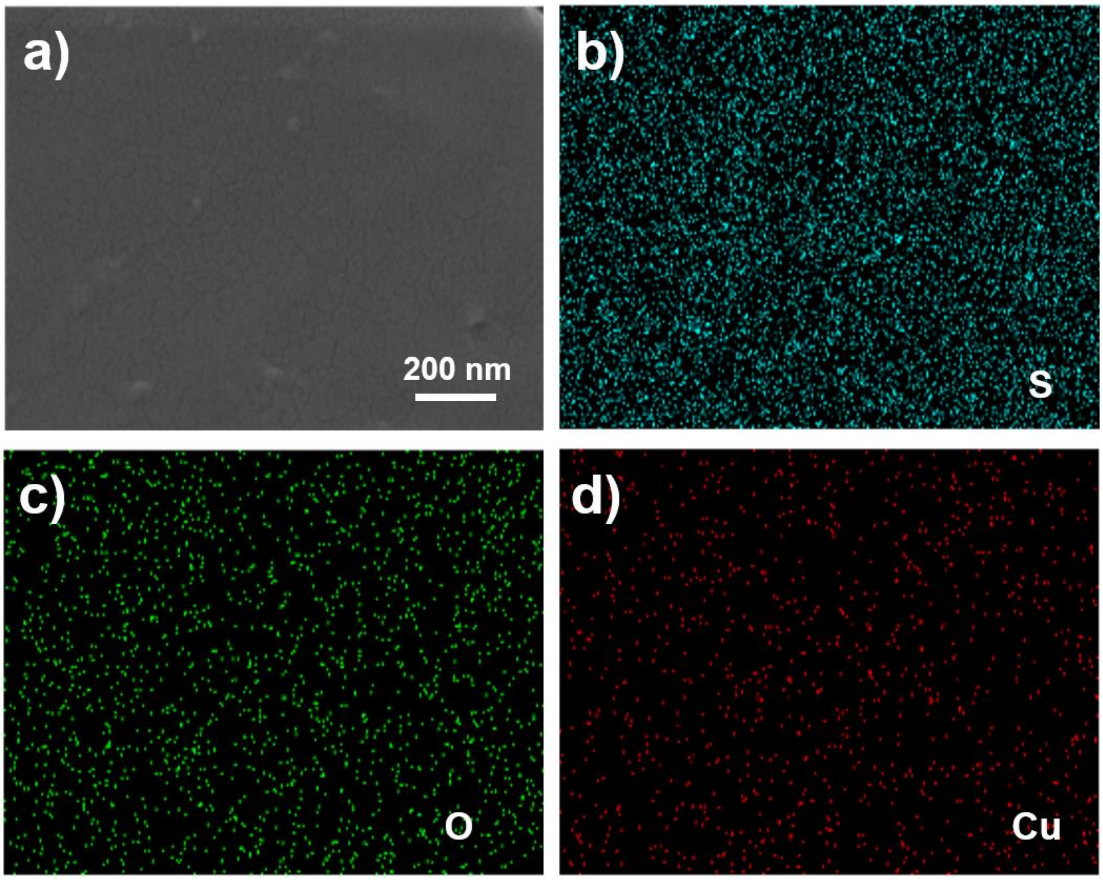

Figure S38. SEM and elemental mapping of HPP-20 after test.

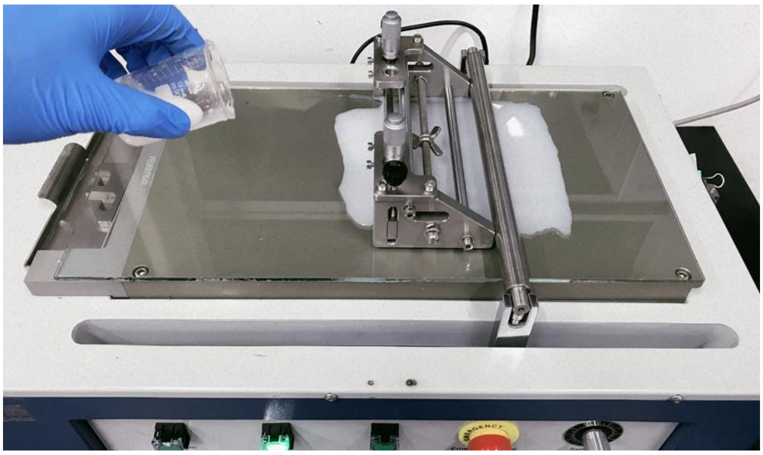

Supplementary Figure 39. The photo image of the casting device for the fabrication of UiO-66@PVDF membrane.

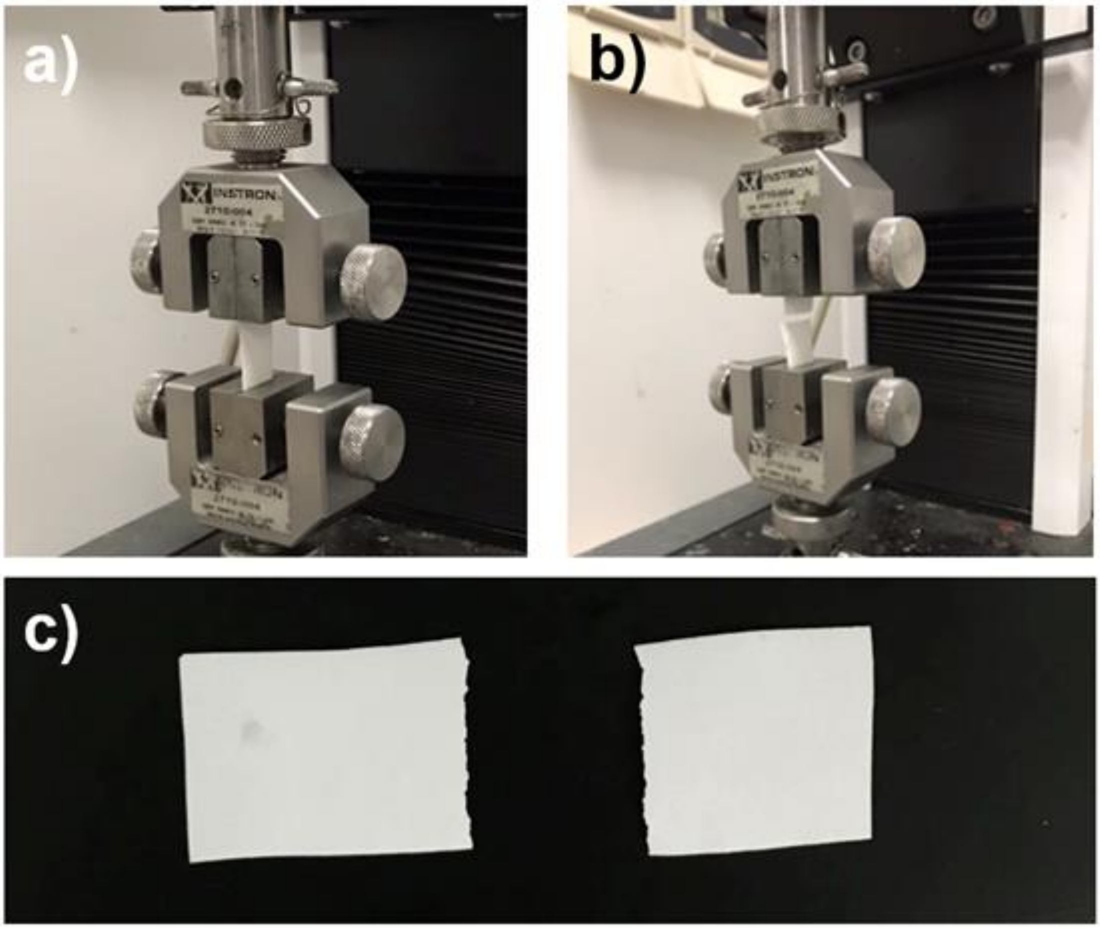

Figure S40. The device of tensile test. The obtained membrane is cut into a strip sample (length, ~5 cm) and (width, ~15 mm). Before the test, the clamp spacing is controlled to be 2-3 cm. The stretching rate is controlled at 5 mm min^{-1} for most of samples except for the PP based ones (10 mm min^{-1}). a) Before test. b) After test. c) Example of strip after test.

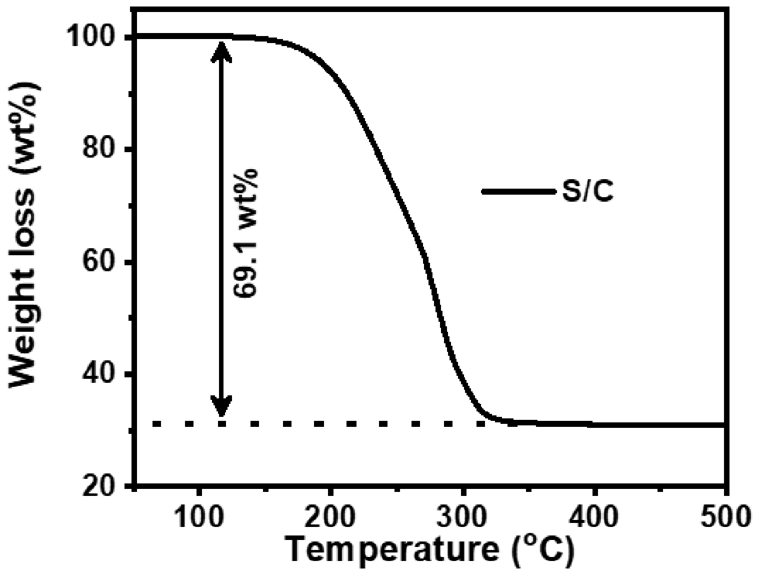

Figure S41. TGA curves of S/C composite under N_2 atmosphere with heating rate at 5 $^{o}C\ min^{-1}$.

Reference

[1] M. S. Denny, Jr., M. Kalaj, K. C. Bentz, S. M. Cohen, *Chem. Sci.* **2018**, *9*, 8842.

[2] S. Foley, H. Geaney, G. Bree, K. Stokes, S. Connolly, M. J. Zaworotko, K. M. Ryan, *Adv. Funct. Mater.* **2018**, *28*, 1800587.

[3] L. Valenzano, B. Civalleri, S. Chavan, S. Bordiga, M. H. Nilsen, S. Jakobsen, K. P. Lillerud, C. Lamberti, *Chem. Mater.* **2011**, *23*, 1700.

[4] M. Mozafari, R. Abedini, A. Rahimpour, *J. Mater. Chem. A* **2018**, *6*, 12380.

[5] Y. Zang, F. Pei, J. Huang, Z. Fu, G. Xu, X. Fang, *Adv. Energy Mater.* **2018**, *8*, 1802052.

[6] Y. Fan, Z. Niu, F. Zhang, R. Zhang, Y. Zhao, G. Lu, *ACS Omega* **2019**, *4*, 10328.

[7] S. Bai, X. Liu, K. Zhu, S. Wu, H. Zhou, *Nat. Energy* **2016**, *1*, 16094.

[8] S. Bai, K. Zhu, S. Wu, Y. Wang, J. Yi, M. Ishida, H. Zhou, *J. Mater. Chem. A* **2016**, *4*, 16812.

[9] Y. He, Z. Chang, S. Wu, Y. Qiao, S. Bai, K. Jiang, P. He, H. Zhou, *Adv. Energy Mater.* **2018**, *8*, 1802130.

[10] Y. Guo, M. Sun, H. Liang, W. Ying, X. Zeng, Y. Ying, S. Zhou, C. Liang, Z. Lin, X. Peng, *ACS Appl. Mater. Interfaces* **2018**, *10*, 30451.

[11] M. Li, Y. Wan, J.-K. Huang, A. H. Assen, C.-E. Hsiung, H. Jiang, Y. Han, M. Eddaoudi, Z. Lai, J. Ming, L.-J. Li, *ACS Energy Lett.* **2017**, *2*, 2362.

[12] D. H. Lee, J. H. Ahn, M.-S. Park, A. Eftekhari, D.-W. Kim, *Electrochim. Acta* **2018**, *283*, 1291.

[13] X. J. Hong, C. L. Song, Y. Yang, H. C. Tan, G. H. Li, Y. P. Cai, H. Wang, *ACS Nano* **2019**, *13*, 1923.

[14] F. Wu, S. Zhao, L. Chen, Y. Lu, Y. Su, Y. Jia, L. Bao, J. Wang, S. Chen, R.

Chen, *Energy Storage Materials* **2018**, *14*, 383.
[15] D. D. Han, Z. Y. Wang, G. L. Pan, X. P. Gao, *ACS Appl. Mater. Interfaces* **2019**, *11*, 18427.
[16] H. Chen, Y. Xiao, C. Chen, J. Yang, C. Gao, Y. Chen, J. Wu, Y. Shen, W. Zhang, S. Li, F. Huo, B. Zheng, *ACS Appl. Mater. Interfaces* **2019**, *11*, 11459.
[17] M. Tian, F. Pei, M. Yao, Z. Fu, L. Lin, G. Wu, G. Xu, H. Kitagawa, X. Fang, *Energy Storage Mater.* **2019**, *21*, 14.
[18] S. H. Kim, J. S. Yeon, R. Kim, K. M. Choi, H. S. Park, *J. Mater. Chem. A* **2018**, *6*, 24971.
[19] Z. Chang, Y. Qiao, J. Wang, H. Deng, P. He, H. Zhou, *Energy Storage Mater.* **2020**, *25*, 164.
[20] S. S. Zhang, *ChemElectroChem* **2020**, DOI: 10.1002/celc.202000650.
